# Converting microwave and telecom photons with a silicon photonic nanomechanical interface

**DOI:** 10.1038/s41467-020-18269-z

**Published:** 2020-09-08

**Authors:** G. Arnold, M. Wulf, S. Barzanjeh, E. S. Redchenko, A. Rueda, W. J. Hease, F. Hassani, J. M. Fink

**Affiliations:** 1 Institute of Science and Technology Austria, Am Campus 1, 3400 Klosterneuburg, Austria; 2 Present Address: Institute for Quantum Science and Technology (IQST), University of Calgary, Calgary, AB Canada

**Keywords:** Optomechanics, Superconducting devices, Quantum optics, Quantum information

## Abstract

Practical quantum networks require low-loss and noise-resilient optical interconnects as well as non-Gaussian resources for entanglement distillation and distributed quantum computation. The latter could be provided by superconducting circuits but existing solutions to interface the microwave and optical domains lack either scalability or efficiency, and in most cases the conversion noise is not known. In this work we utilize the unique opportunities of silicon photonics, cavity optomechanics and superconducting circuits to demonstrate a fully integrated, coherent transducer interfacing the microwave X and the telecom S bands with a total (internal) bidirectional transduction efficiency of 1.2% (135%) at millikelvin temperatures. The coupling relies solely on the radiation pressure interaction mediated by the femtometer-scale motion of two silicon nanobeams reaching a *V*_*π*_ as low as 16 μV for sub-nanowatt pump powers. Without the associated optomechanical gain, we achieve a total (internal) pure conversion efficiency of up to 0.019% (1.6%), relevant for future noise-free operation on this qubit-compatible platform.

## Abstract (web summary)

Existing solutions to interface the microwave and optical domains lack either scalability or efficiency. Here, the authors demonstrate a CMOS compatible converter between microwave and optical signals based on silicon an optomechanical device with a total bidirectional transduction efficiency of 1.2% at millikelvin temperatures.

## Introduction

Large scale quantum networks will facilitate the next level in quantum information technology, such as the internet did for classical communication, enabling, e.g., secure communication and distributed quantum computation^1^. Some of the most promising platforms to process quantum information locally, such as superconducting circuits^2^, spins in solids^3^, and quantum dots^4^, operate naturally in the gigahertz frequency range, but the long-distance transmission of gigahertz radiation is relatively lossy and not resilient to ambient noise. This limits the length of supercooled microwave waveguides in a realistic scenario to tens of meters^5^. In contrast, the transport of quantum information over distances of about 100 km is nowadays routinely achieved by sending optical photons at telecom frequency through optical fibers.

There is a variety of platforms, which in principle have shown to be able to merge the advantages of both worlds ranging from mechanical, piezoelectric, electro-optic, magneto-optic, rare-earth, and Rydberg atom implementations^6,7^. So far, the optomechanical approach^8^ has been proven to be most efficient, reaching a record high photon conversion efficiency of up to 47% with an added noise photon number of only 38^9^. But this composite device is based on a Fabry–Perot cavity that has to be hand assembled and utilizes a membrane mode that is restricted to relatively low mechanical frequencies. Using piezo-electricity, coherent conversion between microwave and optical frequencies has been shown uni- and bidirectional at room temperature^10,11^, and at low temperatures^12,13^ with integrated devices, so far with either low efficiency or unspecified conversion noise properties. The electro-optic platform has shown promising photon conversion efficiencies^14^, recently up to 2% at 2 K^15^, but generally requires very large pump powers in the milliwatt range^16^.

In this work we present a device that converts coherent signals between 10.5 GHz and 198 THz at millikelvin temperatures via the radiation pressure interaction. Due to the need of only picowatt range pump powers, both the heat load to the cryostat and local heating of the integrated device is minimized. The chip-scale device is fabricated from CMOS compatible materials on a commercial silicon-on-insulator wafer over an area of ~200 μm × 120 μm. It is compact, versatile and fully compatible with silicon photonics^17^ and superconducting qubits^18^. The unique electro-opto-mechanical design is optimized for very strong field confinements and radiation pressure couplings, which enable internal efficiencies exceeding unity for ultra-low pump powers. We find that the conversion noise so far precludes a quantum limited operation and we present a comprehensive theoretical and experimental noise analysis to evaluate the potential for scalable and noise-free conversion in the future. Such a power-efficient, ultra-sensitive, and highly integrated hybrid interconnect might find applications ranging from quantum communication^8^ and RF receivers^19^ to magnetic resonance imaging^20^.

## Results

### Transducer theory

**Fig. 1:**
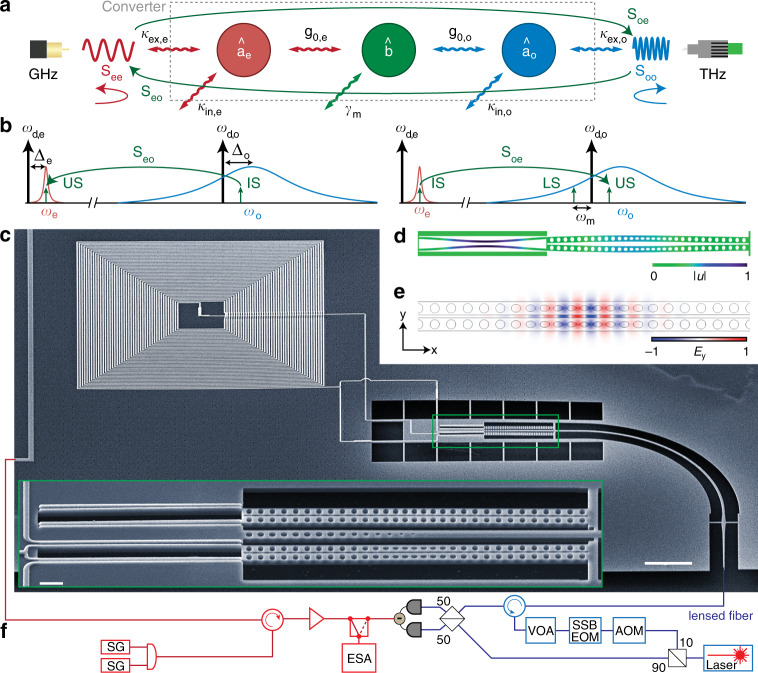
Silicon photonic microwave-to-optics converter. **a** Diagram showing the microwave $({\hat{a}}_{{\rm{e}}})$, mechanical $(\hat{b})$, and optical $({\hat{a}}_{{\rm{o}}})$ mode, and the relevant coupling and loss rates of the device. Scattering parameters *S*_ij_ characterizing the transducer performance are indicated. **b** Schematic showing the frequencies of the coherent signals involved in the conversion process (green arrows). On the left an optical input signal (IS) is converted to an upper sideband (US) of the microwave pump signal, whereas in the microwave-to-optics conversion on the right also the lower sideband (LS) is created. The bidirectional transduction *ζ* is only evaluated between the upper sidebands at *ω*_d,j_ + *ω*_m_. **c** Scanning electron micrograph of the device showing the microwave lumped element resonator with an inductively coupled feed line, the photonic crystal cavity, and the optical coupling waveguide fabricated on a fully suspended 220 nm thick silicon-on-insulator device layer. White scale bar, 10 μm. The inset shows an enlarged view of the central part (green boxed area) comprising the mechanically compliant vacuum gap capacitors of size  ~70 nm and two optomechancial zipper cavities (top one unused) with a central tapered photonic crystal mirror coupled optical waveguide. White scale bar, 1 μm. **d** Finite-element method (FEM) simulation of the mechanical displacement ∣*u*∣ of the utilized mechanical resonance. **e** FEM simulation of the electric in-plane-field *E*_y_(*x*, *y*) for the relevant optical mode. **f** Simplified experimental setup. The device is mounted on the mixing chamber plate of a cryogen-free dilution refrigerator at a temperature of *T*_fridge_ = 50 mK. A microwave switch selects between the incoming microwave and optical signal to be analyzed by the ESA. Optical heterodyning is used to detect the low power levels used in the experiment. SG microwave signal generator; ESA electronic spectrum analyzer; VOA variable optical attenuator; SSB EOM single-sideband electro-optic modulator; AOM acousto-optic modulator.

The transducer consists of one microwave resonator and one optical cavity, both parametrically coupled via the vacuum coupling rates *g*_0,j_ with *j* = *e*, *o* to the same mechanical oscillator as shown in Fig. 1a and b. The intrinsic decay rate of the optical (microwave) resonator is *κ*_in,o_ (*κ*_in,e_), while the optical (microwave) waveguide–resonator coupling is given by *κ*_ex,o_ (*κ*_ex,e_) resulting in a total damping rate of *κ*_j_ = *κ*_in,j_ + *κ*_ex,j_ and coupling ratios *η*_j_ = *κ*_ex,j_/*κ*_j_. The mechanical oscillator with intrinsic decoherence rate *γ*_m_ and frequency *ω*_m_ is shared between the optical cavity and the microwave resonator and acts as a bidirectional coherent pathway to convert the photons between the two different frequencies^8,21–23^. In the interaction frame, the Hamiltonian describing the conversion process is (see Supplementary Note 1):

1 $${\hat{H}}_{{\rm{int}}}=\sum _{\mathrm{j} = {\rm{e}},{\rm{o}}}\left(\hslash {G}_{\mathrm{j}}({\hat{a}}_{\mathrm{j}}^{\dagger }\hat{b}+{\hat{a}}_{\mathrm{j}}{\hat{b}}^{\dagger })+{\hat{H}}_{{\rm{CR}},\mathrm{j}}\right),$$

where ${\hat{a}}_{\mathrm{j}}$, $(\hat{b})$ with *j* = *e*, *o* is the annihilator operator of the electromagnetic (mechanical) mode, and ${\hat{H}}_{{\rm{CR}},\mathrm{j}}=\hslash {G}_{\mathrm{j}}({\hat{a}}_{\mathrm{j}}\hat{b}\ {e}^{2i{\omega }_{{\rm{m}}}t}+\,\text{h.c.})$ describes the counter-rotating terms which are responsible for the coherent amplification of the signal. ${G}_{\mathrm{j}}=\sqrt{{n}_{{\rm{d}},\mathrm{j}}}{g}_{0,\mathrm{j}}$ is the parametrically enhanced electro- or optomechanical coupling rate where *n*_d,j_ is the intracavity photon number due to the corresponding microwave and optical pump tones. For a red-detuned drive in the resolved-sideband regime 4*ω*_m_ > *κ*_j_ we neglect ${\hat{H}}_{{\rm{CR}},\mathrm{j}}$ under the rotating-wave approximation and the Hamiltonian (1) represents a beam-splitter like interaction in which the mechanical resonator mediates noiseless photon conversion between microwave and optical modes. Note that near-unity photon conversion ${\zeta }_{{\rm{RS}}}=4{\eta }_{{\rm{e}}}{\eta }_{{\rm{o}}}{{\mathcal{C}}}_{{\rm{e}}}{{\mathcal{C}}}_{{\rm{o}}}/{(1+{{\mathcal{C}}}_{{\rm{e}}}+{{\mathcal{C}}}_{{\rm{o}}})}^{2}$ can be achieved in the limit of ${{\mathcal{C}}}_{{\rm{e}}}={{\mathcal{C}}}_{{\rm{o}}}\gg 1$ with ${{\mathcal{C}}}_{\mathrm{j}}=4{G}_{\mathrm{j}}^{2}/({\kappa }_{\mathrm{j}}{\gamma }_{{\rm{m}}})$ being the electro- or optomechanical cooperativity, as demonstrated between two optical^24^ and two microwave modes^25,26^, respectively.

### Transducer design

We realize conversion by connecting an optomechanical photonic crystal zipper cavity^27^ with two aluminum coated and mechanically compliant silicon nanostrings^28^ as shown in Fig. 1c. The mechanical coupling between these two components is carefully designed (see Supplementary Note 2), leading to a hybridization of their in-plane vibrational modes into symmetric and antisymmetric supermodes. In case of the antisymmetric mode that is used in this experiment, the strings and the photonic crystal beams vibrate 180° out of phase as shown by the finite-element method simulation in Fig. 1d. The photonic crystal cavity features two resonances at telecom frequencies with similar optomechanical coupling strength. The simulated spatial distribution of the electric field component *E*_y_(*x*, *y*) of the higher frequency mode with lower loss rate used in the experiment is shown in Fig. 1e. The lumped element microwave resonator consists of an ultra-low stray capacitance planar spiral coil inductor^29^ and two mechanically compliant capacitors with a vacuum gap of size of ~70 nm. This resonator is inductively coupled to a shorted coplanar waveguide, which is used to send and retrieve microwave signals from the device. The sample is fabricated using a robust multi-step recipe including electron beam lithography, silicon etching, aluminum thin-film deposition, and hydrofluoric vapor acid etching, as described in detail in ref. ^30^.

### Transducer characterization

Standard sample characterization (see Supplementary Notes 3 and 4) reveals an optical resonance frequency of *ω*_o_/(2*π*) = 198.081 THz with total loss rate *κ*_o_/(2*π*) = 1.6 GHz and waveguide coupling rate *κ*_ex,o_/(2*π*) = 0.18 GHz leading to a coupling efficiency of *η*_o_ = 0.11. When the optical light is turned off, the microwave resonance frequency is *ω*_e_/(2*π*) = 10.5 GHz with coupling efficiency *η*_e_ = 0.4 and *κ*_ex,e_/(2*π*) = 1.15 MHz. The mechanical resonator frequency has a value of *ω*_m_/(2*π*) = 11.843 MHz with an intrinsic decoherence rate *γ*_m_/(2*π*) = 15 Hz at a mode temperature of 150 mK. The achieved single-photon-phonon coupling rates are as high as *g*_0,e_/(2*π*) = 67 Hz and *g*_0,o_/(2*π*) = 0.66 MHz.

### Conversion measurements

**Fig. 2:**
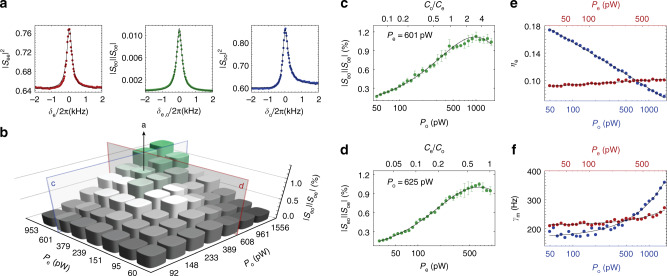
Coherent scattering parameter measurements. **a** The reflection ∣*S*_jj_∣^2^ (*j* = *e*, *o*) and bidirectional transduction *ζ*: = ∣*S*_eo_∣∣*S*_oe_∣ parameters as a function of signal tone detuning *δ*_j_ = *ω* − (*ω*_d,j_ + *ω*_m_) for fixed pump powers *P*_e_ = 601 pW and *P*_o_ = 625 pW. The dots represent the experimental data while the solid lines show the theoretical prediction with *γ*_m_ as the only fit parameter. **b** Measured photon number transduction efficiency as a function of microwave and optical pump powers. **c** Measured transduction efficiency with respect to optical pump power for fixed *P*_e_ = 601 pW. **d** Measured transduction efficiency with respect to microwave pump power for fixed *P*_o_ = 625 pW. In (**c**) and (**d**), the error bars are the standard deviation of three independent measurement runs and solid lines are theory with interpolated *γ*_m_ (from (**f**)) and no other free parameters. **e** The coupling efficiency of the microwave resonator to the waveguide *η*_e_ = *κ*_ex,e_/*κ*_e_, extracted from broadband reflection measurements, as a function of optical (blue, *P*_e_ = 601 pW) and microwave (red, *P*_o_ = 625 pW) pump tones. **f** Intrinsic mechanical decoherence rate *γ*_m_ versus optical (blue, *P*_e_ = 601 pW) and microwave (red, *P*_o_ = 625 pW) pump powers extracted from (**c**) and (**d**) with Eq. (3) and *γ*_m_ as only fit parameter. Solid lines in (**f**) are linear fits to the data.

To perform coherent photon conversion, red-detuned microwave and optical tones with powers *P*_e(o)_ are applied to the microwave and the optical resonator. These drive tones establish the linearized electro- and optomechanical interactions, which results in the conversion of a weak microwave (optical) signal tone to the optical (microwave) domain measured in our setup as shown in Fig. 1f. We experimentally characterize the transducer efficiency by measuring the normalized reflection ∣*S*_jj_∣^2^ (*j* = *e*, *o*) and the bidirectional transmission *ζ* : = ∣*S*_eo_∣∣*S*_oe_∣ coefficients as a function of signal detuning *δ*_*j*_. As shown in Fig. 2a, for drive powers *P*_e_ = 601 pW and *P*_o_ = 625 pW with drive frequencies *ω*_d,j_ and detunings Δ_j_ = *ω*_j_ − *ω*_d,j_ of Δ_e_ = *ω*_m_ and Δ_o_/(2*π*) = 126  MHz leading to intracavity photon numbers of *n*_d,e_ $\approx$ 9 × 10^5^ and *n*_d,o_ $\approx$ 0.2 with cooperativities ${{\mathcal{C}}}_{{\rm{e}}}\approx 0.57$ and ${{\mathcal{C}}}_{{\rm{o}}}\approx 0.9$, the measured total (waveguide to waveguide) photon transduction efficiency is $\approx$1.1% corresponding to 96.7% internal (resonator to resonator) photon transduction efficiency over the total bandwidth of Γ_conv_/(2*π*) $\approx$ 0.37 kHz. In the case of *κ*_o_ > 4*ω*_m_ and *κ*_e_ < 4*ω*_m_, the bandwidth is given by ${\Gamma }_{{\rm{conv}}}\approx ({{\mathcal{C}}}_{{\rm{e}}}+1){\gamma }_{{\rm{m}}}$ because the nonsideband resolved optomechanical cavity does not induce mechanical broadening. The signal tone adds 17(10^−3^) photons to the microwave resonator (optical cavity).

Here we use a self-calibrated measurement scheme that is independent of the gain and loss of the measurement lines as described in ref. ^31^ and we only take into account transduction between the upper two sidebands at *ω*_d,j_ + *ω*_m_ as shown in Fig. 1b. Neglecting the lower optical sideband that is generated due to the nonsideband resolved situation *κ*_o_/4*ω*_m_ $\approx$ 30 reduces the reported mean bidirectional efficiencies by $\sqrt{2}$ compared to the actually achieved total transduction efficiency between microwave and optical fields. The observed reflection peaks indicate that both resonators are undercoupled, equivalent to an impedance mismatch for incoming signal light. All scattering parameters are obtained from measured coherent tones whose linewidths are given by the chosen resolution bandwidth and the stability of the heterodyne setup. While this does not explicitly show long term phase stability of the conversion we find that these results are in excellent agreement with our coherent conversion theory model (solid lines) with *γ*_m_ as the only free fit parameter.

Figure 2b shows the total transduction efficiency for different pump power combinations with microwave and optical pump powers ranging from 30 to 953 pW and 48 to 1561 pW, respectively. Figure 2c, d shows the efficiency versus *P*_o_ (*P*_e_) for fixed microwave (optical) pump power *P*_e_ = 601 (*P*_o_ = 625) pW. As expected, the transduction efficiency rises with increasing pump powers and reaches a maximum of *ζ* = 1.2%. The internal transduction efficiency is significantly higher (*ζ*/(*η*_o_*η*_e_) ≤ 135%) because both the microwave resonator as well as the optical cavity are highly undercoupled with coupling ratios of *η*_o_ = 0.11 and *η*_e_ ranging between 0.07 and 0.18 when both pumps are on. The increase in the intrinsic loss rate of microwave *κ*_in,e_ and mechanical resonator *γ*_m_ at higher pump powers are shown in Fig. 2e and f caused by considerable heating related to (especially optical) photon absorption. This results in the degradation of the microwave and mechanical quality factors and consequently reduces the waveguide coupling efficiency, the cooperativities and the total transduction efficiency (see Supplementary Note 5).

### Sideband resolution and amplification

In the nonsideband resolved limit the contribution of the counter-rotating term of the Hamiltonian ${\hat{H}}_{{\rm{CR}},{\rm{o}}}$ is nonnegligible, resulting in a transduction process that cannot be fully noise- free. This interesting effect can be correctly described by introducing an amplification of the signal tone with (in the absence of thermal noise) quantum limited gain ${{\mathcal{G}}}_{{\rm{o}}}$ (see Supplementary Note 1). In contrast, the microwave resonator is in the resolved-sideband condition 4*ω*_m_ > *κ*_e_, so that the signal tone amplification due to electromechanical interaction is negligible ${{\mathcal{G}}}_{{\rm{e}}}\simeq 1$. This results in the total, power independent, bidirectional conversion gain of ${\mathcal{G}}={{\mathcal{G}}}_{{\rm{e}}}{{\mathcal{G}}}_{{\rm{o}}}\simeq {{\mathcal{G}}}_{{\rm{o}}}$, which turns out to be directly related to the minimum reachable phonon occupation:

2 $${\langle n\rangle }_{\min }=\frac{{({\Delta }_{{\rm{o}}}-{\omega }_{{\rm{m}}})}^{2}+{\kappa }_{{\rm{o}}}^{2}/4}{4{\Delta }_{{\rm{o}}}{\omega }_{{\rm{m}}}}={{\mathcal{G}}}_{{\rm{o}}}-1,$$

induced by optomechanical quantum backaction when the mechanical resonator is decoupled from its thermal bath^32^. Due to this amplification process the measured transduction efficiency in Fig. 2a is about 110 times larger than one would expect from a model that does not include gain effects for the chosen detuning, and adds the equivalent of at least one half of a vacuum noise photon to the input of the transducer in our case of heterodyne detection (for *η*_*j*_ = 1 and ${\mathcal{G}}\gg 1$). However, it turns out that this noise limitation, which might in principle be overcome with efficient feedforward^9^, sideband suppression^33,34^, or sideband resolution^35^, accounts for only about 0.1% of the total conversion noise observed in our system. The total transduction (including gain) can be written in terms of the susceptibilities of the electromagnetic modes ${\chi }_{\mathrm{j}}^{-1}(\omega )=i({\Delta }_{\mathrm{j}}-\omega )+{\kappa }_{\mathrm{j}}/2$ and the mechanical resonator ${\chi }_{{\rm{m}}}^{-1}(\omega )=i({\omega }_{{\rm{m}}}-\omega )+{\gamma }_{{\rm{m}}}/2$ as:

3 $$\zeta ={\left|\frac{\sqrt{{\kappa }_{{\rm{ex}},{\rm{e}}}{\kappa }_{{\rm{ex}},{\rm{o}}}}{G}_{{\rm{e}}}{G}_{{\rm{o}}}{\chi }_{{\rm{e}}}{\chi }_{{\rm{o}}}\left[-{\chi }_{{\rm{m}}}+{\tilde{\chi }}_{{\rm{m}}}\right]}{1+[{\chi }_{{\rm{m}}}-{\tilde{\chi }}_{{\rm{m}}}]\left[{G}_{{\rm{e}}}^{2}({\chi }_{{\rm{e}}}-{\tilde{\chi }}_{{\rm{e}}})+{G}_{{\rm{o}}}^{2}({\chi }_{{\rm{o}}}-{\tilde{\chi }}_{{\rm{o}}})\right]}\right|}^{2},$$

where $$\tilde{{\chi }_{\mathrm{j}}}(\omega )={\chi }_{\mathrm{j}}{(-\omega )}^{* }$$.

**Fig. 3:**
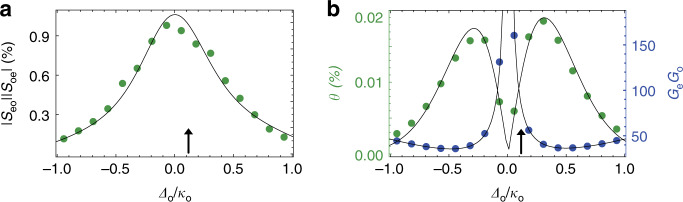
Pure and amplified conversion. **a** Measured total transduction efficiency *ζ*: = ∣*S*_eo_∣∣*S*_oe_∣ with respect to pump detuning Δ_o_ of the nonsideband resolved optical cavity for a constant intracavity photon number *n*_d,o_ = 0.185. **b** Pure and—in the absence of thermal noise—noiseless conversion *θ* (green dots) as well as the total conversion gain ${\mathcal{G}}$ (blue dots) that gives rise to amplified vacuum noise. *θ* is extracted from (**a**) by dividing the measured total transduction by the calculated gain using Supplementary Eqs. (16) and (17). While ${{\mathcal{G}}}_{{\rm{o}}}$ diverges for Δ_o_ → 0, the optomechanical damping rate drops to zero and leads to a vanishing pure conversion *θ*. The arrows in both panels indicate the detuning used in Figs. 2 and 4.

Equation (3) can be decomposed into a product of the conversion gain ${\mathcal{G}}$ and the pure conversion efficiency *θ*, i.e., $\zeta :={\mathcal{G}}\times \theta$, for frequencies in the vicinity of *ω*_m_ (see Supplementary Note 1). Equation (2) shows that the signal amplification depends only on the resonator linewidth and the detuning and is not directly related to the $\propto {\hat{a}}^{\dagger }{\hat{b}}^{\dagger }$ interaction term or the pump power^31^. This can be understood by the alternative interpretation that the gain represents the ratio of the transduced upper sideband to the difference between upper and lower sideband at each cavity. Therefore, it is instructive to measure the transducer parameters as a function of optical pump detuning as shown in Fig. 3a. While changing the optical detuning, we also vary the pump power in order to keep the optical intracavity photon number constant at *n*_d,o_ = 0.185 ± 0.015. This way it is possible to investigate the influence of Δ_o_ at a constant optomechanical coupling ${G}_{{\rm{o}}}={g}_{{\rm{0,o}}}\sqrt{{n}_{{\rm{d}},{\rm{o}}}}$. The measured total transduction efficiency is shown in Fig. 3a and reaches  ζ$\,\approx\,$1% at Δ_o_ $\approx$ 0 for the chosen pump powers in agreement with Fig. 2c, d. We can now separate the measured transduction (Eq. (3)) into conversion gain and pure conversion, as shown in Fig. 3b. The gain shows the expected steep increase at Δ_o_ → 0 where the pure conversion *θ* approaches zero for equal cooling and amplification rates. Around Δ_o_ = *κ*_o_/2 on the other hand, where ${\langle n\rangle }_{\min }$ reaches its minimum of roughly *κ*_o_/4*ω*_m_ $\approx$ 30, also the gain reaches its minimum and the noiseless part (at zero temperature) of the total (internal) conversion process shows its highest efficiency of *θ* = 0.019% (*θ*/(*η*_e_*η*_o_) = 1.6%).

### Added noise

**Fig. 4:**
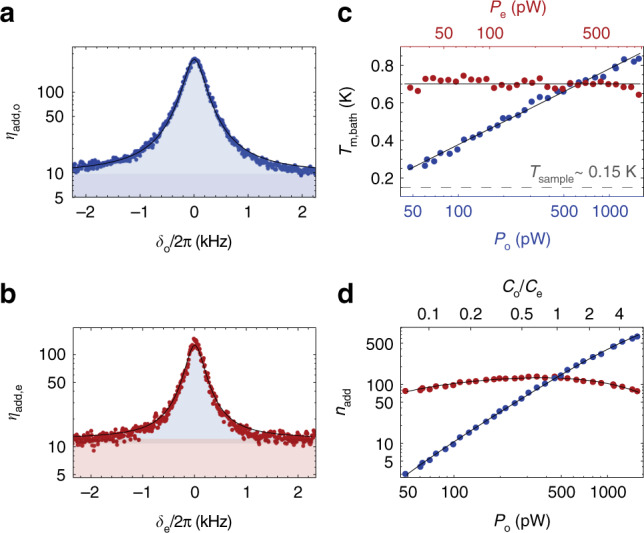
Conversion noise properties. Measured noise spectra at the device output for the optical cavity (**a**) and the microwave resonator (**b**) as a function of the signal tone detuning *δ*_j_ = *ω* − (*ω*_d,j_ + *ω*_m_) at fixed pump powers *P*_e_ = 601 pW and *P*_o_ = 625 pW in units of added noise quanta. **a** The dark blue region represents the two-quadrature noise added by the optical measurement chain; the light blue region indicates the thermal mechanical noise added to the converted optical output signal. **b** Bottom light red region represents the two-quadrature background noise from the microwave measurement chain. The central dark red region indicates a small amount of broadband resonator noise and the light blue region the transduction noise due to the thermal population of the mechanical mode. In both panels, fits to one common mechanical bath *n*_m_ are shown in black. **c** Mechanical bath temperature *T*_m,bath_ extracted as only fit parameter from fits to the measured output noise as in (**a**) and (**b**) with respect to optical (blue dots, *P*_e_ = 601 pW) and microwave (red dots, *P*_o_ = 625 ± 19 pW) pump power. Black lines show fits to the data with the logarithmic growth function $T_{\mathrm{m,bath}}=0.18\ {\mathrm{log}\,}_{{\rm{e}}}({P}_{{\rm{o}}})-0.47$ and *T*_m,bath_ = 0.70 K respectively. The dashed line indicates the thermalized mechanical mode temperature *T*_sample_ when both pumps are off. **d** Microwave (red, *n*_add,e_) and optical (blue, *n*_add,o_) added noise photons at the output with respect to optical pump power (*P*_e_ = 601 pW). The full theory based on an interpolation of *T*_m,bath_ from (**c**) is shown as black lines.

Another important figure of merit, not only for quantum applications, is the amount of added noise quanta^36^, usually an effective number referenced to the input of the device. For clarity with regards to the physical origin and the actual measurement of the noise power, in the following we define the total amount of added noise quanta *n*_add,j_ added to the input signal *S*_in,j_ after the transduction process as *S*_out,j_ = *ζS*_in,j_ + *n*_add,j_. Figure 4a, b shows the measured conversion noise *n*_add,j_ as a function of frequency *δ*_j_ for the same powers and detunings as in Fig. 2a. At these powers our device adds *n*_add,o(e)_ = 224(145) noise quanta to the output of the microwave resonator (optical cavity), corresponding to an effective input noise of *n*_add,j_/*ζ*. The noise floor originates from the calibrated measurement system and in case of the microwave port to a small part also from an additional broadband resonator noise, cf. Fig. 4b. The solid lines are fits to the theory with the mechanical bath occupation ${\bar{n}}_{{\rm{m}}}$ as the only fit parameter (see Supplementary Note 4).

The fitted effective mechanical bath temperature as a function of pump powers is shown in Fig. 4c. It reveals the strong optical pump dependent mechanical mode heating (blue), while the microwave pump (red) has a negligible influence on the mechanical bath. Fig. 4d shows the measured total added noise at the output of the microwave resonator and optical cavity as a function of optical pump power. The noise added to the optical output (blue) increases with pump power due to absorption heating and increasing optomechanical coupling rate *G*_o_, while the degradation of the resonator-waveguide coupling efficiency *η*_e_ explains the decreasing *n*_add,e_ at higher optical powers for the microwave output noise (red), see Fig. 2e. The intersection of the two noise curves occurs at ${{\mathcal{C}}}_{{\rm{e}}}\simeq {{\mathcal{C}}}_{{\rm{o}}}$ with cooperativities *C*_j_ as defined above, and shows that the optical and microwave resonators share the same mechanical thermal bath. The power dependence is in full agreement with theory (solid lines) and demonstrates that the thermal mechanical population is the dominating origin of the added transducer noise.

## Discussion

In conclusion, we demonstrated an efficient bidirectional and chip-scale microwave-to-optics transducer using pump powers orders of magnitude lower than comparable all-integrated^11,13,15^ approaches. Low pump powers are desired to limit the heat load of the cryostat and to minimize on-chip heating, which is particularly important for integrated devices because of their limited heat dissipation at millikelvin temperatures. Due to the standard material choice involving only silicon and aluminum, our device can be easily integrated with other elements of superconducting circuits as well as silicon photonic and phononic devices in the future.

The two main challenges ahead are the reduced pure conversion efficiency and the optical heating that adds incoherent noise to the converted signal. We expect that both can be solved with design improvements in combination with new measurement techniques. Specifically, starting from the observed pure efficiency of 0.019% a factor of up to nearly two orders of magnitude could be gained with better waveguide coupling geometries in combination with fabrication optimization, e.g., by using surface cleaning and the reduction of humidity^37^. Improving the sideband resolution by increasing the mechanical frequency^35^ could yield another factor of up to 25 assuming the same cooperativities can be achieved. Going to the high cooperativity limit would then yield the remaining fraction needed for unity total conversion efficiency. This will certainly require a very effective mitigation of the optical pump power dependent mechanical heating and the associated linewidth degradation that is also required for noise-free conversion. Nevertheless, with better chip thermalization, reduced optical absorption and low duty cycle pulsed measurements this should be feasible. Moreover, it has already been shown that pulsed pump-probe type experiments together with high efficiency heralding measurements can be used for postselecting rare successful conversion or entanglement generation events for low-noise low-efficiency devices^12,38^.

In terms of near-term classical receiver and modulation applications, an important figure of merit is the voltage required to induce an optical phase shift of *π*. We are able to reach a value as low as *V*_π_ = 16 μV (see Supplementary Note 6), comparable with typical zero point fluctuations in superconducting circuits, nearly a factor 9 lower than the previously reported record^19^, and almost 10^12^ times more power efficient than commercial passive and wide-band unidirectional electro-optic modulators at X band gigahertz frequencies.

## Supplementary information

Supplementary Information

Peer Review File

## Data Availability

The data and code used to produce the results of this paper are available at 10.5281/zenodo.3961562.
