# Publisher Correction: Converting microwave and telecom photons with a silicon photonic nanomechanical interface

**DOI:** 10.1038/s41467-020-18912-9

**Published:** 2020-10-01

**Authors:** G. Arnold, M. Wulf, S. Barzanjeh, E. S. Redchenko, A. Rueda, W. J. Hease, F. Hassani, J. M. Fink

**Affiliations:** 1 Institute of Science and Technology Austria, Am Campus 1, 3400 Klosterneuburg, Austria; 2 Present Address: Institute for Quantum Science and Technology (IQST), University of Calgary, Calgary, AB Canada

**Keywords:** Optomechanics, Superconducting devices, Quantum optics, Quantum information

Correction to: *Nature Communications* 10.1038/s41467-020-18269-z, published online 8 September 2020.

The original version of this Article contained errors in the author affiliations. J. M. Fink was incorrectly associated with ‘Institute for Quantum Science and Technology (IQST), University of Calgary, Calgary, AB, Canada’ in the HTML version of the paper.

Additionally, the present address of the author S. Barzanjeh, ‘Institute for Quantum Science and Technology (IQST), University of Calgary, Calgary, AB, Canada’ was incorrectly assigned as a full affiliation in the HTML version of the paper.

These errors have been corrected in the HTML version of the Article. The PDF was correct at the time of publication.

