# Supplementary material for: Converting microwave and telecom photons with a silicon photonic nanomechanical interface
Source: Nat Commun. 2020 Sep 8;11:4460. doi: 10.1038/s41467-020-18269-z (PMC7479601; doi:10.1038/s41467-020-18269-z)
Supplement: Supplementary file 1 — Supplementary Information [file 41467_2020_18269_MOESM1_ESM.pdf]

Supplementary Information for: "Converting microwave and telecom photons with a silicon photonic nanomechanical interface"

G. Arnold,[1,*] M. Wulf,[1,*] S. Barzanjeh,[1,†] E. S. Redchenko,[1] A. Rueda,[1] W. J. Hease,[1] F. Hassani,[1] and J. M. Fink[1,‡]

[1] *Institute of Science and Technology Austria, Am Campus 1, 3400 Klosterneuburg, Austria*

(Dated: July 27, 2020)

CONTENTS

Page

Supplementary Note 1. Theoretical model of the microwave-optical converter 2
- A Hamiltonian . . . 2
- B Equations of motion . . . 2
- C Conversion efficiency and gain . . . 4
- D Conversion bandwidth . . . 5
- E Added noise . . . 6

Supplementary Note 2. Device design 7
- A Optical cavity . . . 7
- B LC circuit . . . 7
- C Electro- and optomechanical coupling . . . 9
- D Mechanical oscillator . . . 9
- E Device parameter summary . . . 10

Supplementary Note 3. Experimental setup 10

Supplementary Note 4. Characterization 12
- A Resonator measurements . . . 12
- B Calibration . . . 12
  - 1 Microwave measurement system . . . 12
  - 2 Optical measurement system . . . 14
- C Conversion noise and heating . . . 16
  - 1 Microwave pump . . . 16
  - 2 Microwave resonator . . . 16
  - 3 Detuning dependencies . . . 17
- D Bidirectionality and calibration uncertainties . . . 17

Supplementary Note 5. Decomposition of the transducer performance 19

Supplementary Note 6. Performance as a classical phase modulator 20
- A Modulation voltage V_π . . . 20
- B Estimate for the energy-per-bit E_{bit} . . . 21
- C Comparison of the values of V_π and E_{bit} to literature . . . 21

References 23

* Authors contributed equally.
† Present address: Institute for Quantum Science and Technology (IQST), University of Calgary, Canada.
‡ jfink@ist.ac.at

Supplementary Note 1. THEORETICAL MODEL OF THE MICROWAVE-OPTICAL CONVERTER

A. Hamiltonian

Our electro-optomechanical system consists of a mechanical resonator with resonance frequency ω_{m} that is capacitively coupled to a superconducting microwave resonator and optically to a photonic crystal cavity, as shown in Fig. 1(a) and (b) of the main text. The microwave resonator and optical cavity are driven using microwave and laser pump tones with frequencies $\omega_{\mathrm{d},j} = \omega_j - \Delta_j$, where Δ_j are the detunings from their resonant frequencies ω_j, with $j =$ e,o. We include intrinsic losses for the microwave resonator and optical cavity with rates $\kappa_{\mathrm{in},j}$, and use $\kappa_{\mathrm{ex},j}$ to denote their input-port coupling rates. The Hamiltonian of the coupled system is given by [1]

$$\hat{H} = \hbar\omega_{\mathrm{m}}\hat{b}^\dagger\hat{b} + \hbar\sum_{j=\mathrm{e,o}}\Big[\omega_j\hat{c}_j^\dagger\hat{c}_j + g_{0,j}(\hat{b}^\dagger + \hat{b})\hat{c}_j^\dagger\hat{c}_j + iE_j(\hat{c}_j^\dagger e^{-i\omega_{\mathrm{d},j}t} - \hat{c}_j e^{i\omega_{\mathrm{d},j}t})\Big], \tag{1}$$

where $\hat{b}$ is the annihilation operator of the mechanical resonator, $\hat{c}_j$ is the annihilation operator for resonator $j =$ e,o whose coupling rate to the mechanical resonator is $g_{0,j}$. The microwave/optical driving strength for resonator j is $E_j = \sqrt{\kappa_{\mathrm{ex},j}\, P_j/\hbar\omega_{\mathrm{d},j}}$, where P_j is the power of the driving field [2].

In the interaction frame with respect to $\hbar\sum_{j=\mathrm{e,o}}\omega_{\mathrm{d},j}c_j^\dagger c_j$ and neglecting terms oscillating at $\pm2\omega_{\mathrm{d},j}$, the system Hamiltonian reduces to

$$\hat{H} = \hbar\omega_{\mathrm{m}}\hat{b}^\dagger\hat{b} + \hbar\sum_{j=\mathrm{e,o}}\Big[\Delta_j + g_{0,j}(\hat{b}^\dagger + \hat{b})\Big]\hat{c}_j^\dagger\hat{c}_j + \hat{H}_{\mathrm{d}}, \tag{2}$$

where the Hamiltonian associated with the driving fields is $\hat{H}_{\mathrm{d}} = i\hbar\sum_{j=\mathrm{e,o}}E_j(\hat{c}_j^\dagger - \hat{c}_j)$.

We can linearize Hamiltonian (2) by expanding the microwave and optical modes around their steady-state field amplitudes, $\hat{a}_j = \hat{c}_j - \sqrt{n_{\mathrm{d},j}}$, where $n_{\mathrm{d},j} = |E_j|^2/(\kappa_j^2/4 + \Delta_j^2)$ is the mean number of intra-cavity photons induced by the microwave and optical pumps [2], $\kappa_j = \kappa_{\mathrm{in},j} + \kappa_{\mathrm{ex},j}$ are the total resonator decay rates, and Δ_j are the effective resonator and cavity detunings. The linearized Hamiltonian becomes

$$\hat{H} = \hbar\omega_{\mathrm{m}}\hat{b}^\dagger\hat{b} + \hbar\sum_{j=\mathrm{e,o}}\Big[\Delta_j\hat{a}_j^\dagger\hat{a}_j + G_j(\hat{b} + \hat{b}^\dagger)(\hat{a}_j^\dagger + \hat{a}_j)\Big], \tag{3}$$

where $G_j = g_{0,j}\sqrt{n_{\mathrm{d},j}}$. By setting the effective resonator detunings so that $\Delta_{\mathrm{e}} = \Delta_{\mathrm{o}} = \omega_{\mathrm{m}}$, moving to an interaction frame, and neglecting the terms rotating at $\pm2\omega_{\mathrm{m}}$, the above Hamiltonian reduces to

$$\hat{H} = \hbar G_{\mathrm{e}}(\hat{a}_{\mathrm{e}}\hat{b}^\dagger + \hat{b}\hat{a}_{\mathrm{e}}^\dagger) + \hbar G_{\mathrm{o}}(\hat{a}_{\mathrm{o}}\hat{b}^\dagger + \hat{b}\hat{a}_{\mathrm{o}}^\dagger), \tag{4}$$

as specified in the main text.

B. Equations of motion

The full quantum treatment of the system can be given in terms of the quantum Langevin equations in which we add to the Heisenberg equations the quantum noise acting on the mechanical resonator ($\hat{b}_{\mathrm{in}}$ with damping rate γ_m), as well as the resonator and cavity input fluctuations ($\hat{a}_{\mathrm{ex},j}$, for $j =$ e,o, with rates $\kappa_{\mathrm{ex},j}$), plus the intrinsic losses of the resonator and cavity modes ($\hat{a}_{\mathrm{in},j}$, for $j =$ e,o, with loss rates $\kappa_{\mathrm{in},j}$). These noises have the correlation functions

$$\langle\hat{a}_{\mathrm{ext},j}(t)\hat{a}_{\mathrm{ext},j}^\dagger(t')\rangle = \langle\hat{a}_{\mathrm{ext},j}^\dagger(t)\hat{a}_{\mathrm{ext},j}(t')\rangle + \delta(t-t') = (\bar{n}_{\mathrm{ext},j} + 1)\delta(t-t'), \tag{5a}$$

$$\langle\hat{a}_{\mathrm{in},j}(t)\hat{a}_{\mathrm{in},j}^\dagger(t')\rangle = \langle\hat{a}_{\mathrm{in},j}^\dagger(t)\hat{a}_{\mathrm{in},j}(t')\rangle + \delta(t-t') = (\bar{n}_{\mathrm{in},j} + 1)\delta(t-t'), \tag{5b}$$

$$\langle\hat{b}_{\mathrm{in}}(t)\hat{b}_{\mathrm{in}}^\dagger(t')\rangle = \langle\hat{b}_{\mathrm{in}}^\dagger(t)\hat{b}_{\mathrm{in}}(t')\rangle + \delta(t-t') = (\bar{\mathrm{n}}_m + 1)\delta(t-t'), \tag{5c}$$

where $\bar{n}_{\mathrm{ext},j}$, $\bar{n}_{\mathrm{in},j}$, and $\bar{n}_{\mathrm{m}}$ are the Planck-law thermal occupancies of each bath with $j =$ e,o. The resulting Langevin equations corresponding to Hamiltonian (3) are

$$\dot{\hat{a}}_j = -(\frac{\kappa_j}{2} + i\Delta_j)\hat{a}_j - iG_j(\hat{b} + \hat{b}^\dagger) + \sqrt{\kappa_{\mathrm{ext},j}}\hat{a}_{\mathrm{ex},j} + \sqrt{\kappa_{\mathrm{in},j}}\hat{a}_{\mathrm{in},j}, \tag{6a}$$

$$\dot{\hat{b}} = -(\frac{\gamma_{\mathrm{m}}}{2} + i\omega_{\mathrm{m}})\hat{b} - i\sum_{j=\mathrm{e,o}}G_j(\hat{a}_j + \hat{a}_j^\dagger) + \sqrt{\gamma_{\mathrm{m}}}\hat{b}_{\mathrm{in}}. \tag{6b}$$

We can solve the above equations in the Fourier domain to obtain the microwave resonator and optical cavity variables. By substituting the solutions of Eqs. (6a)–(6b) into the corresponding input-output relation, i.e., $\hat{a}_{\text{out},j} = \sqrt{\kappa_{\text{ex},j}}\hat{a}_j - \hat{a}_{\text{ex},j}$, we obtain

$$\mathbf{S}_{\text{out}}(\omega) = \mathbf{\Upsilon}(\omega)\mathbf{S}_{\text{in}}(\omega), \tag{7}$$

where $\mathbf{\Upsilon}(\omega) = \Big(\mathbf{C}.[-i\omega\mathbf{I} - \mathbf{A}]^{-1}.\mathbf{B} - \mathbf{D}\Big)$ with $\mathbf{I}$ is the identity matrix, $\mathbf{S}_{\text{out}} = [\hat{a}_{\text{out,e}}, \hat{a}_{\text{out,o}}, \hat{a}^{\dagger}_{\text{out,e}}, \hat{a}^{\dagger}_{\text{out,o}}]^T$, $\mathbf{S}_{\text{in}} = [\hat{a}_{\text{ext,e}}, \hat{a}_{\text{in,e}}, \hat{a}_{\text{ext,o}}, \hat{a}_{\text{in,o}}, \hat{b}_{\text{in}}, \hat{a}^{\dagger}_{\text{ext,e}}, \hat{a}^{\dagger}_{\text{in,e}}, \hat{a}^{\dagger}_{\text{ext,o}}, \hat{a}^{\dagger}_{\text{in,o}}, \hat{b}^{\dagger}_{\text{in}}]^T$, and we have defined the following matrices

$$\mathbf{A} = \begin{bmatrix} -(\frac{\kappa_{\text{e}}}{2} + i\Delta_{\text{e}}) & 0 & -iG_{\text{e}} & 0 & 0 & -iG_{\text{e}} \\ 0 & -(\frac{\kappa_{\text{o}}}{2} + i\Delta_{\text{o}}) & -iG_{\text{o}} & 0 & 0 & -iG_{\text{o}} \\ -iG_{\text{e}} & -iG_{\text{o}} & -(\frac{\gamma_{\text{m}}}{2} + i\omega_{\text{m}}) & -iG_{\text{e}} & -iG_{\text{o}} & 0 \\ 0 & 0 & iG_{\text{e}} & -(\frac{\kappa_{\text{e}}}{2} - i\Delta_{\text{e}}) & 0 & iG_{\text{e}} \\ 0 & 0 & iG_{\text{o}} & 0 & -(\frac{\kappa_{\text{o}}}{2} - i\Delta_{\text{o}}) & iG_{\text{o}} \\ iG_{\text{e}} & iG_{\text{o}} & 0 & iG_{\text{e}} & iG_{\text{o}} & -(\frac{\gamma_{\text{m}}}{2} - i\omega_{\text{m}}) \end{bmatrix}, \tag{8}$$

$$\mathbf{B} = \begin{bmatrix} \sqrt{\kappa_{\text{e}}\eta_{\text{e}}} & \sqrt{\kappa_{\text{e}}(1-\eta_{\text{e}})} & 0 & 0 & 0 & 0 & 0 & 0 & 0 & 0 \\ 0 & 0 & \sqrt{\kappa_{\text{o}}\eta_{\text{o}}} & \sqrt{\kappa_{\text{o}}(1-\eta_{\text{o}})} & 0 & 0 & 0 & 0 & 0 & 0 \\ 0 & 0 & 0 & 0 & \sqrt{\gamma_{\text{m}}} & 0 & 0 & 0 & 0 & 0 \\ 0 & 0 & 0 & 0 & 0 & \sqrt{\kappa_{\text{e}}\eta_{\text{e}}} & \sqrt{\kappa_{\text{e}}(1-\eta_{\text{e}})} & 0 & 0 & 0 \\ 0 & 0 & 0 & 0 & 0 & 0 & 0 & \sqrt{\kappa_{\text{o}}\eta_{\text{o}}} & \sqrt{\kappa_{\text{o}}(1-\eta_{\text{o}})} & 0 \\ 0 & 0 & 0 & 0 & 0 & 0 & 0 & 0 & 0 & \sqrt{\gamma_{\text{m}}} \end{bmatrix}, \tag{9}$$

$$\mathbf{C} = \begin{bmatrix} \sqrt{\kappa_{\text{e}}\eta_{\text{e}}} & 0 & 0 & 0 & 0 & 0 \\ 0 & \sqrt{\kappa_{\text{o}}\eta_{\text{o}}} & 0 & 0 & 0 & 0 \\ 0 & 0 & 0 & \sqrt{\kappa_{\text{e}}\eta_{\text{e}}} & 0 & 0 \\ 0 & 0 & 0 & 0 & \sqrt{\kappa_{\text{o}}\eta_{\text{o}}} & 0 \end{bmatrix}, \tag{10}$$

$$\mathbf{D} = \begin{bmatrix} 1 & 0 & 0 & 0 & 0 & 0 & 0 & 0 & 0 & 0 \\ 0 & 0 & 1 & 0 & 0 & 0 & 0 & 0 & 0 & 0 \\ 0 & 0 & 0 & 0 & 0 & 1 & 0 & 0 & 0 & 0 \\ 0 & 0 & 0 & 0 & 0 & 0 & 0 & 1 & 0 & 0 \end{bmatrix}, \tag{11}$$

with $\eta_j = \kappa_{\text{ext,j}}/\kappa_j$. The total output fields are then

$$\begin{aligned} \hat{a}_{\text{out,e}} = (\eta_{\text{e}}\alpha_{\text{e,e}} - 1)\hat{a}_{\text{ext,e}} + \sqrt{\eta_{\text{e}}}\Big[&\sqrt{1-\eta_{\text{e}}}\alpha_{\text{e,e}}\hat{a}_{\text{int,e}} + \sqrt{\eta_{\text{o}}}\alpha_{\text{e,o}}\hat{a}_{\text{ext,o}} + \sqrt{1-\eta_{\text{o}}}\alpha_{\text{e,o}}\hat{a}_{\text{int,o}} + \alpha_{\text{e,m}}\hat{b}_{\text{in}} \\ &+\sqrt{\eta_{\text{e}}}\tilde{\alpha}_{\text{e,e}}\hat{a}^{\dagger}_{\text{ext,e}} + \sqrt{1-\eta_{\text{e}}}\tilde{\alpha}_{\text{e,e}}\hat{a}^{\dagger}_{\text{int,e}} + \sqrt{\eta_{\text{o}}}\tilde{\alpha}_{\text{e,o}}\hat{a}^{\dagger}_{\text{ext,o}} + \sqrt{1-\eta_{\text{o}}}\tilde{\alpha}_{\text{e,o}}\hat{a}^{\dagger}_{\text{int,o}} + \tilde{\alpha}_{\text{e,m}}\hat{b}^{\dagger}_{\text{in}}\Big], \end{aligned} \tag{12a}$$

$$\begin{aligned} \hat{a}_{\text{out,o}} = (\eta_{\text{o}}\alpha_{\text{o,o}} - 1)\hat{a}_{\text{ext,o}} + \sqrt{\eta_{\text{o}}}\Big[&\sqrt{1-\eta_{\text{o}}}\alpha_{\text{o,o}}\hat{a}_{\text{int,o}} + \sqrt{\eta_{\text{e}}}\alpha_{\text{o,e}}\hat{a}_{\text{ext,e}} + \sqrt{1-\eta_{\text{e}}}\alpha_{\text{o,e}}\hat{a}_{\text{int,e}} + \alpha_{\text{o,m}}\hat{b}_{\text{in}} \\ &+\sqrt{\eta_{\text{o}}}\tilde{\alpha}_{\text{o,o}}\hat{a}^{\dagger}_{\text{ext,o}} + \sqrt{1-\eta_{\text{o}}}\tilde{\alpha}_{\text{o,o}}\hat{a}^{\dagger}_{\text{int,o}} + \sqrt{\eta_{\text{e}}}\tilde{\alpha}_{\text{o,e}}\hat{a}^{\dagger}_{\text{ext,e}} + \sqrt{1-\eta_{\text{e}}}\tilde{\alpha}_{\text{o,e}}\hat{a}^{\dagger}_{\text{int,e}} + \tilde{\alpha}_{\text{o,m}}\hat{b}^{\dagger}_{\text{in}}\Big], \end{aligned} \tag{12b}$$

with $\hat{a}^{(\dagger)}_{\mathrm{ext},j}$ and $\hat{a}^{(\dagger)}_{\mathrm{int},j}$ referring to modes in the waveguide and bath respectively and the coefficients

$$\alpha_{\mathrm{e,e}} = \frac{\kappa_\mathrm{e}\,\chi_\mathrm{e}\Big(1+G_\mathrm{o}^2(\chi_\mathrm{o}-\chi_\mathrm{o}^*)-G_\mathrm{e}^2\chi_\mathrm{e}^*\Big)\Big[-\chi_\mathrm{m}+\chi_\mathrm{m}^*\Big]}{1+[\chi_\mathrm{m}-\chi_\mathrm{m}^*]\big[G_\mathrm{e}^2(\chi_\mathrm{e}-\chi_\mathrm{e}^*)+G_\mathrm{o}^2(\chi_\mathrm{o}-\chi_\mathrm{o}^*)\big]}, \tag{13a}$$

$$\alpha_{\mathrm{o,o}} = \frac{\kappa_\mathrm{o}\,\chi_\mathrm{o}\Big(1+G_\mathrm{e}^2(\chi_\mathrm{e}-\chi_\mathrm{e}^*)-G_\mathrm{o}^2\chi_\mathrm{o}^*\Big)\Big[-\chi_\mathrm{m}+\chi_\mathrm{m}^*\Big]}{1+[\chi_\mathrm{m}-\chi_\mathrm{m}^*]\big[G_\mathrm{e}^2(\chi_\mathrm{e}-\chi_\mathrm{e}^*)+G_\mathrm{o}^2(\chi_\mathrm{o}-\chi_\mathrm{o}^*)\big]}, \tag{13b}$$

$$\alpha_{\mathrm{e,o}} = \alpha_{\mathrm{o,e}} = \frac{\sqrt{\kappa_\mathrm{e}\kappa_\mathrm{o}}\,\chi_\mathrm{e}\chi_\mathrm{o}G_\mathrm{e}G_\mathrm{o}\Big[-\chi_\mathrm{m}+\chi_\mathrm{m}^*\Big]}{1+[\chi_\mathrm{m}-\chi_\mathrm{m}^*]\big[G_\mathrm{e}^2(\chi_\mathrm{e}-\chi_\mathrm{e}^*)+G_\mathrm{o}^2(\chi_\mathrm{o}-\chi_\mathrm{o}^*)\big]}, \tag{13c}$$

$$\alpha_{j,\mathrm{m}} = -\frac{i\,\sqrt{\kappa_j\gamma_\mathrm{m}}\,G_j\chi_j\chi_\mathrm{m}}{1+[\chi_\mathrm{m}-\chi_\mathrm{m}^*]\big[G_\mathrm{e}^2(\chi_\mathrm{e}-\chi_\mathrm{e}^*)+G_\mathrm{o}^2(\chi_\mathrm{o}-\chi_\mathrm{o}^*)\big]}, \tag{13d}$$

$$\tilde{\alpha}_{j,j} = \frac{\kappa_j\,\chi_j\chi_j^*G_j^2\Big[-\chi_\mathrm{m}+\chi_\mathrm{m}^*\Big]}{1+[\chi_\mathrm{m}-\chi_\mathrm{m}^*]\big[G_\mathrm{e}^2(\chi_\mathrm{e}-\chi_\mathrm{e}^*)+G_\mathrm{o}^2(\chi_\mathrm{o}-\chi_\mathrm{o}^*)\big]}, \tag{13e}$$

$$\tilde{\alpha}_{\mathrm{e,o}} = \frac{\sqrt{\kappa_\mathrm{e}\kappa_\mathrm{o}}\,\chi_\mathrm{e}\chi_\mathrm{o}^*G_\mathrm{e}G_\mathrm{o}\Big[-\chi_\mathrm{m}+\chi_\mathrm{m}^*\Big]}{1+[\chi_\mathrm{m}-\chi_\mathrm{m}^*]\big[G_\mathrm{e}^2(\chi_\mathrm{e}-\chi_\mathrm{e}^*)+G_\mathrm{o}^2(\chi_\mathrm{o}-\chi_\mathrm{o}^*)\big]}, \tag{13f}$$

$$\tilde{\alpha}_{\mathrm{o,e}} = -\frac{\sqrt{\kappa_\mathrm{e}\kappa_\mathrm{o}}\,\chi_\mathrm{e}^*\chi_\mathrm{o}G_\mathrm{e}G_\mathrm{o}\Big[-\chi_\mathrm{m}+\chi_\mathrm{m}^*\Big]}{1+[\chi_\mathrm{m}-\chi_\mathrm{m}^*]\big[G_\mathrm{e}^2(\chi_\mathrm{e}-\chi_\mathrm{e}^*)+G_\mathrm{o}^2(\chi_\mathrm{o}-\chi_\mathrm{o}^*)\big]}, \tag{13g}$$

$$\tilde{\alpha}_{j,\mathrm{m}} = -\frac{i\,\sqrt{\kappa_j\gamma_\mathrm{m}}\,G_j\chi_j\chi_\mathrm{m}^*}{1+[\chi_\mathrm{m}-\chi_\mathrm{m}^*]\big[G_\mathrm{e}^2(\chi_\mathrm{e}-\chi_\mathrm{e}^*)+G_\mathrm{o}^2(\chi_\mathrm{o}-\chi_\mathrm{o}^*)\big]}, \tag{13h}$$

with $j = \mathrm{e,o}$. We also define the individual susceptibilities of the optical cavity and microwave resonator $\chi_j^{-1} = \chi_j(\omega)^{-1} = i(\Delta_j-\omega)+\kappa_j/2$ and the mechanical susceptibility $\chi_\mathrm{m}^{-1} = \chi_\mathrm{m}(\omega)^{-1} = i(\omega_\mathrm{m}-\omega)+\gamma_\mathrm{m}/2$ and $\chi_k^* = \chi_k(-\omega)^*$ $k = \mathrm{e,o,m}$. Note that the commutation relation $[\hat{a}_{\mathrm{out},j}(\omega), \hat{a}^\dagger_{\mathrm{out},j}(\omega')] = \delta(\omega-\omega')$ imposes the following constrains

$$(|\eta_\mathrm{e}\alpha_\mathrm{e,e}-1|^2+\eta_\mathrm{e}(1-\eta_\mathrm{e})|\alpha_\mathrm{e,e}|^2-\eta_\mathrm{e}|\tilde{\alpha}_\mathrm{e,e}|^2)+\eta_\mathrm{e}(|\alpha_\mathrm{e,o}|^2-|\tilde{\alpha}_\mathrm{e,o}|^2)+\eta_\mathrm{e}(|\alpha_\mathrm{e,m}|^2-|\tilde{\alpha}_\mathrm{e,m}|^2) = 1, \tag{14a}$$

$$(|\eta_\mathrm{o}\alpha_\mathrm{o,o}-1|^2+\eta_\mathrm{o}(1-\eta_\mathrm{o})|\alpha_\mathrm{o,o}|^2-\eta_\mathrm{o}|\tilde{\alpha}_\mathrm{o,o}|^2)+\eta_\mathrm{o}(|\alpha_\mathrm{o,e}|^2-|\tilde{\alpha}_\mathrm{o,e}|^2)+\eta_\mathrm{o}(|\alpha_\mathrm{o,m}|^2-|\tilde{\alpha}_\mathrm{o,m}|^2) = 1. \tag{14b}$$

C. Conversion efficiency and gain

From Eq. (7) we can directly calculate all elements of the scattering matrix including the reflection parameters and transduction efficiency. The *effective* microwave-to-optical transduction efficiency is given by

$$\zeta(\omega) := |\Upsilon_{2,1}|^2 = |\Upsilon_{1,2}|^2 = \Big|\frac{\sqrt{\kappa_{\mathrm{ex,e}}\kappa_{\mathrm{ex,o}}}G_\mathrm{e}G_\mathrm{o}\chi_\mathrm{e}(\omega)\chi_\mathrm{o}(\omega)\Big[-\chi_\mathrm{m}(\omega)+\chi_\mathrm{m}(-\omega)^*\Big]}{1+[\chi_\mathrm{m}(\omega)-\chi_\mathrm{m}(-\omega)^*]\big[G_\mathrm{e}^2(\chi_\mathrm{e}(\omega)-\chi_\mathrm{e}(-\omega)^*)+G_\mathrm{o}^2(\chi_\mathrm{o}(\omega)-\chi_\mathrm{o}(-\omega)^*)\big]}\Big|^2. \tag{15}$$

The above equation contains the pure conversion efficiency and the gain due to the unresolved sideband condition of the optical mode. We can separate these two effects by rewriting Eq. (15) in terms of the electro- and optomechanical damping rates $\Gamma_j = G_j^2\Big[\frac{\kappa_j}{(\Delta_j-\omega)^2+\kappa_j^2/4} - \frac{\kappa_j}{(\Delta_j+\omega)^2+\kappa_j^2/4}\Big]$. This then gives $\zeta(\omega) = \theta\times\mathcal{G}$, where

$$\theta = \Big|\frac{2\sqrt{\eta_\mathrm{e}\eta_\mathrm{o}}\,\sqrt{\Gamma_\mathrm{e}\Gamma_\mathrm{o}}}{2i(\omega-\omega_\mathrm{m}')+\gamma_\mathrm{m}+\Gamma_\mathrm{e}+\Gamma_\mathrm{o}}\Big|^2, \tag{16}$$

is the pure bidirectional optical-to-microwave conversion efficiency with $\omega_\mathrm{m}' = \omega_\mathrm{m}-\delta_\omega(\omega_\mathrm{m})$ and $\delta_\omega(\omega_\mathrm{m}) = \sum_{j=\mathrm{e,o}}\mathrm{Im}(G_j^2(\chi_j^*-\chi_j))$ being the electro- and optomechanical frequency shifts while $\mathcal{G} = \mathcal{G}_\mathrm{o}\mathcal{G}_\mathrm{e}$ is the amplification gain of the converter where

$$\mathcal{G}_\mathrm{e} = \Big(\frac{|\chi_\mathrm{e}|^2}{4\Delta_\mathrm{e}\omega_\mathrm{m}}\Big)\Big[(\Delta_\mathrm{e}-\omega)^2+\kappa_\mathrm{e}^2/4\Big]\Big[(\Delta_\mathrm{e}+\omega)^2+\kappa_\mathrm{e}^2/4\Big], \tag{17a}$$

$$\mathcal{G}_\mathrm{o} = \Big(\frac{|\chi_\mathrm{o}|^2}{4\Delta_\mathrm{o}\omega_\mathrm{m}}\Big)\Big[(\Delta_\mathrm{o}-\omega)^2+\kappa_\mathrm{o}^2/4\Big]\Big[(\Delta_\mathrm{o}+\omega)^2+\kappa_\mathrm{o}^2/4\Big], \tag{17b}$$

are the gains attributed to the unresolved sideband condition of the optical cavity and the microwave resonator. For our system $\delta_\omega \ll \omega_\mathrm{m}$, as such we consider $\omega = \omega'_\mathrm{m} \simeq \omega_\mathrm{m}$, resulting in

$$\mathcal{G}_\mathrm{e} = \Big(\frac{(\Delta_\mathrm{e} + \omega_\mathrm{m})^2 + \kappa_\mathrm{e}^2/4}{4\Delta_\mathrm{e}\,\omega_\mathrm{m}}\Big), \tag{18a}$$

$$\mathcal{G}_\mathrm{o} = \Big(\frac{(\Delta_\mathrm{o} + \omega_\mathrm{m})^2 + \kappa_\mathrm{o}^2/4}{4\Delta_\mathrm{o}\,\omega_\mathrm{m}}\Big). \tag{18b}$$

Note that $\Delta_\mathrm{e} = \omega_\mathrm{m}$ and considering the fact that in our system the microwave resonator is in the resolved sideband regime $\omega_\mathrm{m} \gg \kappa_\mathrm{e}$ entails $\mathcal{G}_\mathrm{e} \simeq 1$. The total gain, therefore, reduces to $\mathcal{G} \simeq \mathcal{G}_\mathrm{o} = 1 + \langle n\rangle_\mathrm{min}$ where

$$\langle n\rangle_\mathrm{min} = \frac{(\Delta_\mathrm{o} - \omega_\mathrm{m})^2 + \kappa_\mathrm{o}^2/4}{4\Delta_\mathrm{o}\omega_\mathrm{m}} \tag{19}$$

is the minimum phonon number of the mechanical resonator induced by the optomechanical quantum backaction when the mechanical resonator is decoupled from its thermal bath [3, 4]. At the optical detuning $\Delta_\mathrm{o} = \kappa_\mathrm{o}/2$ the phononic occupation number at absence of thermal noise reaches its minimum $\langle n\rangle_\mathrm{min} \simeq \kappa_\mathrm{o}/4\omega_\mathrm{m} \gg 1$. In this regime the backaction cooling of the mechanical resonator to its ground state is prohibited.

We can rewrite Eqs. (13) in terms of the system gains

$$\begin{aligned}\eta_\mathrm{e}\eta_\mathrm{o}|\tilde{\alpha}_\mathrm{e,o}|^2 &= \theta\mathcal{G}_\mathrm{e}(\mathcal{G}_\mathrm{o} - 1),\\ \eta_\mathrm{e}\eta_\mathrm{o}|\tilde{\alpha}_\mathrm{o,e}|^2 &= \theta\mathcal{G}_\mathrm{o}(\mathcal{G}_\mathrm{e} - 1),\\ \eta_\mathrm{e}\eta_\mathrm{o}|\alpha_\mathrm{e,o}|^2 &= \eta_\mathrm{e}\eta_\mathrm{o}|\alpha_\mathrm{o,e}|^2 = \theta\mathcal{G}_\mathrm{o}\mathcal{G}_\mathrm{e}.\end{aligned} \tag{20}$$

Using the above equation we can simplify Eqs. (12) to

$$\hat{a}_\mathrm{out,e}/\sqrt{\theta} = \mathcal{G}_\mathrm{e}(\sqrt{\mathcal{G}_\mathrm{o}}\hat{a}_\mathrm{ext,o} + \sqrt{\mathcal{G}_\mathrm{o} - 1}\hat{a}^\dagger_\mathrm{ext,o}) + \sum_{j=e,o}\sum_{i=e,o,m} F_\mathrm{e}(\alpha_{j,i}/\sqrt{\theta}, \hat{O}), \tag{21a}$$

$$\hat{a}_\mathrm{out,o}/\sqrt{\theta} = \mathcal{G}_\mathrm{o}(\sqrt{\mathcal{G}_\mathrm{e}}\hat{a}_\mathrm{ext,e} + \sqrt{\mathcal{G}_\mathrm{e} - 1}\hat{a}^\dagger_\mathrm{ext,e}) + \sum_{j=e,o}\sum_{i=e,o,m} F_\mathrm{o}(\alpha_{j,i}/\sqrt{\theta}, \hat{O}). \tag{21b}$$

The terms inside the brackets on the right hand side of the above equations describe the amplification of the quantum fluctuation $\hat{a}_\mathrm{ext,o(e)}$ at the input port of the optical cavity (microwave resonator) with corresponding gain $\mathcal{G}_\mathrm{o(e)}$ [5]. Here, $F_\mathrm{e(o)}(\alpha_{i,j}/\sqrt{\theta}, \hat{O})$ show the contribution of the quantum fluctuation at the input of the microwave resonator (optical cavity) and mechanical resonator.

At the resonance condition $\omega = \Delta_j = \omega_\mathrm{m}$, the total gain simplifies to $\mathcal{G} = \mathcal{G}_\mathrm{o}\mathcal{G}_\mathrm{e} = [1 + (\kappa_\mathrm{o}/4\omega_\mathrm{m})^2][1 + (\kappa_\mathrm{e}/4\omega_\mathrm{m})^2]$. If electro- and optomechanical cavity are additionally in the resolved sideband regime ($\omega_\mathrm{m} \gg \kappa_j$) all contributions from counter-rotating terms in the Hamiltonian (3) become negligible, resulting in $\mathcal{G} = 1$ and the effective conversion efficiency (15) reduces to

$$\zeta_\mathrm{sbr}(\omega_\mathrm{m}) = \frac{4\eta_\mathrm{e}\eta_\mathrm{o}\Gamma_\mathrm{e}\Gamma_\mathrm{o}}{(\gamma_\mathrm{m} + \Gamma_\mathrm{e} + \Gamma_\mathrm{o})^2} = \frac{4\eta_\mathrm{e}\eta_\mathrm{o}\mathcal{C}_\mathrm{e}\mathcal{C}_\mathrm{o}}{(1 + \mathcal{C}_\mathrm{e} + \mathcal{C}_\mathrm{o})^2}, \tag{22}$$

where Γ_j simplifies to $\Gamma_j = \frac{4g_{0,j}^2 n_{\mathrm{d},j}}{\kappa_j} = \frac{4G_j^2}{\kappa_j} = \mathcal{C}_j\gamma_\mathrm{m}$ with the optomechanical cooperativity $\mathcal{C}_j$.

D. Conversion bandwidth

The bandwidth of the conversion process can be calculated from the denominator of Eq. (15). In our experiment the microwave resonator is in the resolved sideband regime $4\omega_\mathrm{m} \gg \kappa_\mathrm{e}$, while the optical cavity goes beyond this regime $\omega_\mathrm{m} \ll \kappa_\mathrm{o}$. As such for $\omega = \Delta_j = \omega_\mathrm{m} \gg \gamma_\mathrm{m}$ we have $\chi_\mathrm{e} \to 2/\kappa_\mathrm{e}$, $\chi_\mathrm{o} \simeq \chi_\mathrm{o}^* \to 2/\kappa_\mathrm{o}$, $\chi_\mathrm{m} \to 2/\gamma_\mathrm{m}$, and $\{\chi_\mathrm{e}^*, \chi_\mathrm{m}^*\} \to 0$ which gives the following bandwidth

$$\Gamma_\mathrm{conv} \approx \Gamma_\mathrm{e} + \gamma_\mathrm{m}. \tag{23}$$

Its dependence on the optomechanical damping rate $\Gamma_\mathrm{o} \ll \Gamma_\mathrm{e}$ is negligible because the unresolved sideband condition of the optical cavity significantly limits Γ_o due to equal photon scattering to the red and blue sidebands.

E. Added noise

The total noise added during conversion including the vibrational noise of the mechanics and the resonators' noises can be calculated with the spectral density of the output fields

$$2\pi\,\mathbf{S}_{\mathrm{SD}}(\omega)\delta(\omega-\omega') = \langle \mathbf{S}_{\mathrm{out}}(\omega')^{\dagger}\mathbf{S}_{\mathrm{out}}(\omega)\rangle. \tag{24}$$

The input signals for the noise are thermal states with bath occupations $\mathbf{N} = \mathrm{diag}[\bar{n}_{\mathrm{ext,e}}, \bar{n}_{\mathrm{int,e}}, \bar{n}_{\mathrm{ext,o}}, \bar{n}_{\mathrm{int,o}}, \bar{n}_{\mathrm{m}}]$. The spectral density (not symmetrized) can then be written as

$$\mathbf{S}_{\mathrm{SD}}(\omega) = \mathbf{\Upsilon}^{*}(\omega)\mathbf{\Sigma}\mathbf{\Upsilon}^{T}(\omega). \tag{25}$$

with

$$\mathbf{\Sigma} = \begin{bmatrix} \mathbf{N} & 0 \\ 0 & \mathbf{N}+1 \end{bmatrix}. \tag{26}$$

Using Eq. (12), the total noises added to the output of the microwave resonator and optical cavity are given by

$$\begin{aligned} n_{\mathrm{add,e}} = {} & |\eta_{\mathrm{e}}\alpha_{\mathrm{e,e}}-1|^2\bar{n}_{\mathrm{ext,e}} + \eta_{\mathrm{e}}\Big[(1-\eta_{\mathrm{e}})|\alpha_{\mathrm{e,e}}|^2\bar{n}_{\mathrm{int,e}} + \eta_{\mathrm{o}}|\alpha_{\mathrm{e,o}}|^2\bar{n}_{\mathrm{ext,o}} + (1-\eta_{\mathrm{o}})|\alpha_{\mathrm{e,o}}|^2\bar{n}_{\mathrm{int,o}} + |\alpha_{\mathrm{e,m}}|^2\bar{n}_{\mathrm{m}} \\ & + \eta_{\mathrm{e}}|\tilde{\alpha}_{\mathrm{e,e}}|^2(\bar{n}_{\mathrm{ext,e}}+1) + (1-\eta_{\mathrm{e}})|\tilde{\alpha}_{\mathrm{e,e}}|^2(\bar{n}_{\mathrm{int,e}}+1) + \eta_{\mathrm{o}}|\tilde{\alpha}_{\mathrm{e,o}}|^2(\bar{n}_{\mathrm{ext,o}}+1) + (1-\eta_{\mathrm{o}})|\tilde{\alpha}_{\mathrm{e,o}}|^2(\bar{n}_{\mathrm{int,o}}+1) + |\tilde{\alpha}_{\mathrm{e,m}}|^2(\bar{n}_{\mathrm{m}}+1)\Big], \end{aligned} \tag{27a}$$

$$\begin{aligned} n_{\mathrm{add,o}} = {} & |\eta_{\mathrm{o}}\alpha_{\mathrm{o,o}}-1|^2\bar{n}_{\mathrm{ext,o}} + \eta_{\mathrm{o}}\Big[(1-\eta_{\mathrm{o}})|\alpha_{\mathrm{o,o}}|^2\bar{n}_{\mathrm{int,o}} + \eta_{\mathrm{e}}|\alpha_{\mathrm{o,e}}|^2\bar{n}_{\mathrm{ext,e}} + (1-\eta_{\mathrm{e}})|\alpha_{\mathrm{o,e}}|^2\bar{n}_{\mathrm{int,e}} + |\alpha_{\mathrm{o,m}}|^2\bar{n}_{\mathrm{m}} \\ & + \eta_{\mathrm{o}}|\tilde{\alpha}_{\mathrm{o,o}}|^2(\bar{n}_{\mathrm{ext,o}}+1) + (1-\eta_{\mathrm{o}})|\tilde{\alpha}_{\mathrm{o,o}}|^2(\bar{n}_{\mathrm{int,o}}+1) + \eta_{\mathrm{e}}|\tilde{\alpha}_{\mathrm{o,e}}|^2(\bar{n}_{\mathrm{ext,e}}+1) + (1-\eta_{\mathrm{e}})|\tilde{\alpha}_{\mathrm{o,e}}|^2(\bar{n}_{\mathrm{int,e}}+1) + |\tilde{\alpha}_{\mathrm{o,m}}|^2(\bar{n}_{\mathrm{m}}+1)\Big]. \end{aligned} \tag{27b}$$

The noise terms can be simplified in the vacuum condition in which the thermal occupations of the microwave resonator $\bar{n}_{\mathrm{ext,e}} = \bar{n}_{\mathrm{int,e}} = 0$, optical cavity $\bar{n}_{\mathrm{ext,o}} = \bar{n}_{\mathrm{int,o}} = 0$, and mechanical resonator $\bar{n}_{\mathrm{m}} = 0$ are negligible,

$$n_{\mathrm{add,e}} = \eta_{\mathrm{e}}\Big(|\tilde{\alpha}_{\mathrm{e,e}}|^2 + |\tilde{\alpha}_{\mathrm{e,o}}|^2 + |\tilde{\alpha}_{\mathrm{e,m}}|^2\Big), \tag{28a}$$

$$n_{\mathrm{add,o}} = \eta_{\mathrm{o}}\Big(|\tilde{\alpha}_{\mathrm{o,o}}|^2 + |\tilde{\alpha}_{\mathrm{o,e}}|^2 + |\tilde{\alpha}_{\mathrm{o,m}}|^2\Big). \tag{28b}$$

We can write the noise added to the output of the transducer in terms of the electromechanical and optomechanical gain introduced in Eqs. (17). By considering $|\tilde{\alpha}_{\mathrm{e(o),m}}|^2_{\omega=\omega_{\mathrm{m}}} \ll \{|\tilde{\alpha}_{\mathrm{e,o}}|^2, |\tilde{\alpha}_{\mathrm{e,o}}|^2, |\tilde{\alpha}_{j,j}|^2\}$ (since $\chi_{\mathrm{m}}(\omega_{\mathrm{m}}) \gg \chi^{*}_{\mathrm{m}}(-\omega_{\mathrm{m}})$ for $\omega_{\mathrm{m}} \gg \gamma_{\mathrm{m}}$) the Eqs. (28) reduce to

$$n_{\mathrm{add,e}} \simeq \theta/\eta_{\mathrm{o}}\,\mathcal{G}_{\mathrm{e}}\Big[\Big(\frac{\Gamma_{\mathrm{e}}}{\Gamma_{\mathrm{o}}}\Big)(\mathcal{G}_{\mathrm{e}}-1) + (\mathcal{G}_{\mathrm{o}}-1)\Big], \tag{29a}$$

$$n_{\mathrm{add,o}} \simeq \theta/\eta_{\mathrm{e}}\,\mathcal{G}_{\mathrm{o}}\Big[\Big(\frac{\Gamma_{\mathrm{o}}}{\Gamma_{\mathrm{e}}}\Big)(\mathcal{G}_{\mathrm{o}}-1) + (\mathcal{G}_{\mathrm{e}}-1)\Big]. \tag{29b}$$

The above equations can be simplified further and written in terms of the added noises of the optical and microwave amplifier models introduced in Eqs. (21). The resolved sideband condition of either the microwave resonator or optical cavity, respectively, results in $\mathcal{G}_{\mathrm{e}} \simeq 1$ or $\mathcal{G}_{\mathrm{o}} \simeq 1$, as such Eqs. (29) reduce to

$$\begin{aligned} n_{\mathrm{amp,e}} &= \frac{n_{\mathrm{add,e}}}{\theta} \simeq \mathcal{G}_{\mathrm{o}} - 1, \\ n_{\mathrm{amp,o}} &= \frac{n_{\mathrm{add,o}}}{\theta} \simeq \mathcal{G}_{\mathrm{e}} - 1, \end{aligned} \tag{30a}$$

representing the added noise at the output of quantum limited amplifiers with gains $\mathcal{G}_{\mathrm{o}}$ and $\mathcal{G}_{\mathrm{e}}$ considering vacuum noise at the input ports, in agreement with Eqs. (21).

For the special case of $\Delta_e = \omega_m$ and considering the microwave resonator being in the resolved sideband condition i.e. $\mathcal{G}_e \simeq 1$, we can rewrite Eqs. (29) in terms of the phononic occupancy $\langle n \rangle_{\min}$, as

$$n_{\text{add,e}} \simeq \theta/\eta_o\,(\mathcal{G}_o - 1) = \theta/\eta_o\,\langle n \rangle_{\min}, \tag{31a}$$

$$n_{\text{add,o}} \simeq \theta/\eta_e\,(\mathcal{G}_o - 1)\mathcal{G}_o\Big(\frac{\Gamma_o}{\Gamma_e}\Big) = \theta/\eta_e\,\langle n \rangle_{\min}\big(\langle n \rangle_{\min} + 1\big)\Big(\frac{\Gamma_o}{\Gamma_e}\Big). \tag{31b}$$

Note that for $\theta = 1$ and therefore also $\eta_j = 1$, the added noises in Eq. (31)a represents the amplification of the vacuum noise with gain $\mathcal{G}_o$ which is the direct result of the quantum backaction induced phononic occupation $\langle n \rangle_{\min} = \mathcal{G}_o - 1$.

We want to stress that we use microwave and optical heterodyne detection in this experiment. Thus, the outgoing field is detected after interference with a reference beam and the detection is therefore sensitive to emission and absorption of photons from this field [6]. Consequently the measured quantity is correctly described by the single-sided and symmetrized power spectral density given by

$$\mathbf{S}_{\text{SD,sym}}(\omega) = \boldsymbol{\Upsilon}^*(\omega)\boldsymbol{\Sigma}_{\text{sym}}\boldsymbol{\Upsilon}^T(\omega). \tag{32}$$

with

$$\boldsymbol{\Sigma}_{\text{sym}} = \begin{bmatrix} \mathbf{N} + 1/2 & 0 \\ 0 & \mathbf{N} + 1/2 \end{bmatrix}. \tag{33}$$

Supplementary Note 2. DEVICE DESIGN

The transducer device can be divided into the nanomechanical oscillator and the microwave resonator. The nanomechanical oscillator itself consists of the movable string electrodes of the mechanically compliant capacitors of the microwave LC-circuit and the 'zipper'-optomechanical cavity. The design of the string electrodes is based on Refs. [7, 8] whereas the 'zipper'-cavity is inspired by Ref. [9].

The geometry of the device is shown in Supplementary Fig. 1a where all important dimensions are highlighted. The nanomechanical oscillator is surrounded by rectangular cutouts etched in the silicon device layer which act as a buckling shield [7] to reduce the effect of membrane buckling due to compressive stress in the silicon. That would lead to out of plane misalignment of our resonator and therefore to a decrease of $g_{0,o}/(2\pi)$ and $g_{0,e}/(2\pi)$.

A. Optical cavity

The optomechanical 'zipper' cavity was designed in two steps. First, the dimensions of the photonic crystal mirror were determined by FEM simulations (COMSOL multiphysics®) of the unit cell in such a way that there is large bandgap between the first and the second guided band centered around the desired frequency of $\sim 200\,$THz. As a second step, the hole size and the lattice constant were modified to pull up the first mode into the center of the bandgap as shown in Supplementary Fig. 1b. This approach leads to a cavity mode with high quality factor.

The optomechanical 'zipper' cavity is evanescently coupled to a coupling waveguide [10]. Consequently, the distance between cavity and the waveguide determines the strength of the optical waveguide coupling rate $\kappa_{\text{ex,o}}/(2\pi)$. We chose a distance of 400 nm which should lead to a value of 2.15 GHz, according to FEM simulations. This value is comparable to prior experimentally observed internal optical cavity loss rates $\kappa_{\text{in,o}}/(2\pi)$. Unfortunately, we observe a much smaller optical waveguide coupling rate of 0.18 GHz in the fabricated transducer device. The reason for this deviation is subject of future investigations but first FEM simulations indicate fabrication inaccuracies as the cause.

B. LC circuit

The microwave resonator is implemented by means of a LC-circuit. The capacitance is realized by two capacitors connected in parallel, each of them consisting of two aluminum electrodes separated by a $\sim 70\,$nm gap leading to a capacitance of around 0.43 fF according to FEM simulations. Each string of the nanomechanical oscillator acts as one electrode of a capacitor and is therefore mechanically compliant. The inductor is implemented by means of a square coil which consists of 48 turns with a pitch of $0.5\,\mu$m. Its inductance has an analytically calculated value [11] of 59.8 nH. The length of the aluminum wiring between the coil and the capacitors is minimized in order to reduce the stray capacitance of the circuit. Taking into account the simulated values of the two mechanically modulated

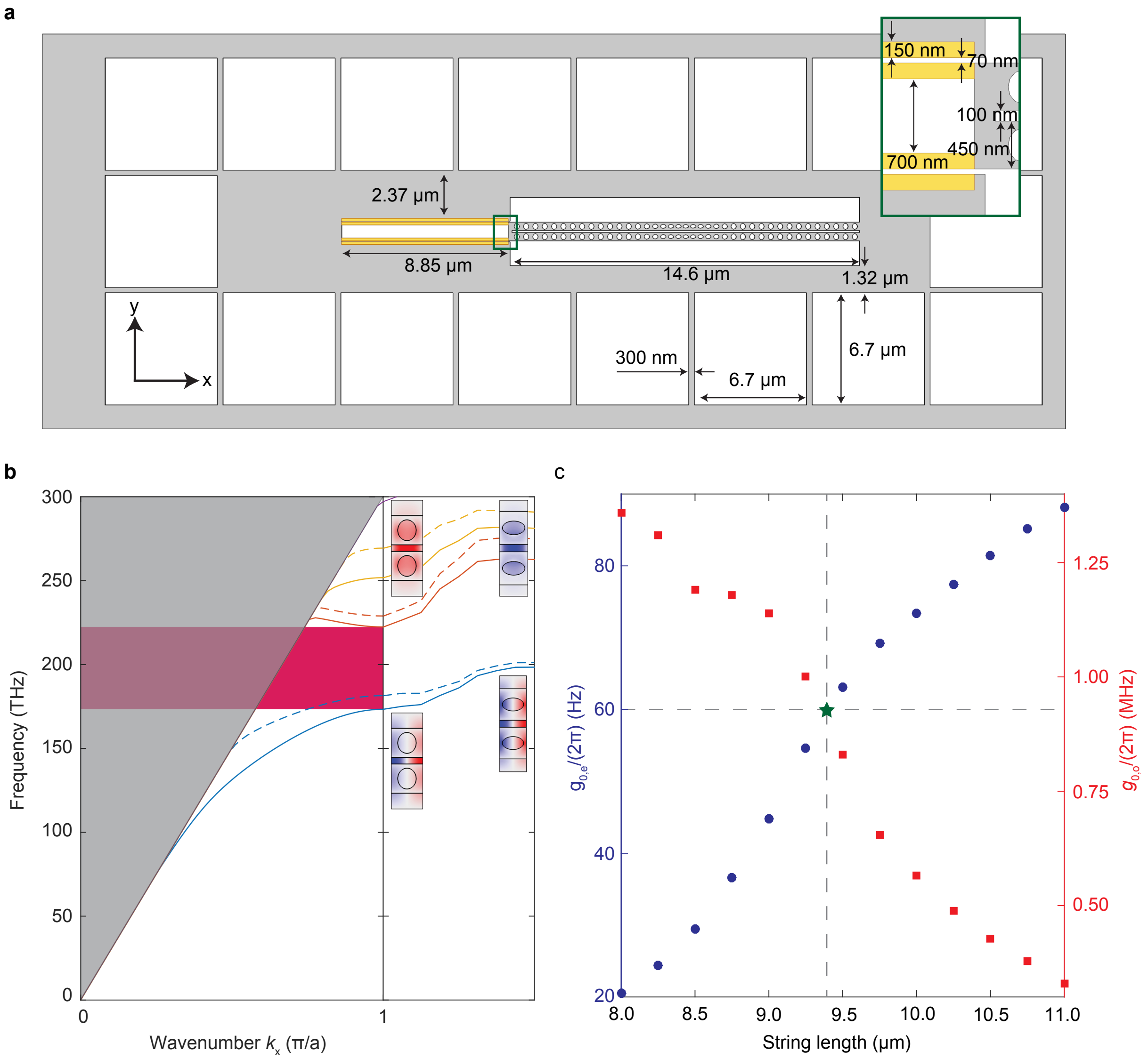

Supplementary Fig. 1. **Device design.** **a**, Geometry of the transducer device. Important dimensions are indicated and the aluminum capacitor electrodes are shown in yellow. Further circuit wiring is not shown. **b**, Photonic band diagram for the optomechanical 'zipper' cavity. The left part of the diagram shows the bands for the mirror region as a function of wavenumber whereas the right part depicts the evolution of the bands towards the cavity defect with a gradual change of hole dimensions. The gray shaded area illustrates the continuum of unguided modes and the pink area highlights the band gap. Solid lines show modes which are symmetric in the y-direction whereas dashed lines show anti-symmetric ones. Insets show the spatial electric field distribution $E_{\mathrm{y}}(x, y)$ for the first and second y-symmetric mode. **c**, Electromechanical (left y-axis) as well as optomechanical (right y-axis) coupling rate extracted from a series of FEM simulations where the string length of the nanomechanical oscillator was swept between 8 and 11 μm. The dashed gray lines indicate the string length chosen for the final design and the corresponding values for $g_{0,\mathrm{e}}/(2\pi)$ and $g_{0,\mathrm{o}}/(2\pi)$.

capacitors with a capacitance of 2×0.43fF, the coil inductance and the measured microwave resonance frequency yields a stray capacity of $C_{\mathrm{s}} \approx 3\,$fF. Our goal was to achieve significant overcoupling for the microwave circuit and we therefore chose the distance of the coil from the microwave feed line to be $9.5\,\mu$m which should have led to an extrinsic coupling rate $\kappa_{\mathrm{ex,e}}/(2\pi)$ of 0.9 MHz, a value above the typical intrinsic losses that we experienced in prior experiments for similar structures. However, due to the heating mechanisms described in the main text in Fig. 4, we are undercoupled, in particular when turning on the optical pump (see Fig. 2e in the main text).

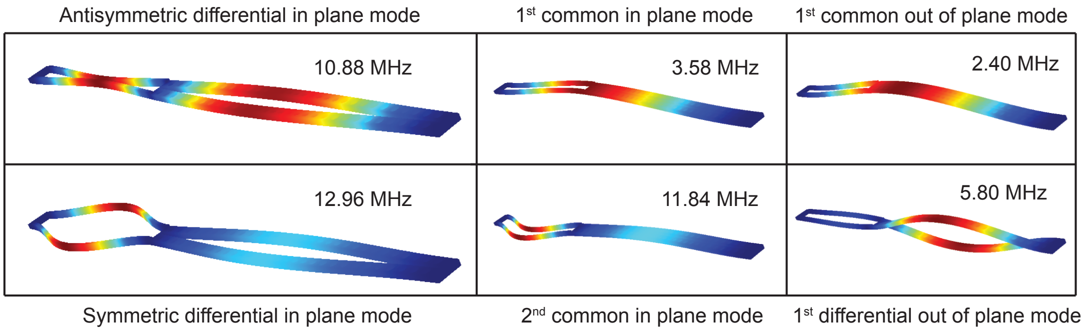

Supplementary Fig. 2. **Simulation of the most relevant mechanical modes.** FEM simulation of the mechanical displacement and the resonance frequency of the first fundamental modes of our nanomechanical oscillator.

C. Electro- and optomechanical coupling

Since the properties of the mechanical motion of the nanomechanical oscillator highly depend on its geometry, its design has to be optimized to reach the best electro- and optomechanical coupling rates. As first step, we choose the length of the optical cavity according to two criteria: 1. the number of mirror cells had to be large enough to reach a decent intrinsic quality factor (in this experiment a value of 1.3×10^5 equivalent to an intrinsic cavity loss rate of $\kappa_{\mathrm{in,o}}/(2\pi) = 1.42\,\mathrm{GHz}$) and 2. the total length had to be short enough so that we could keep the thermal occupation at the fridge base temperature relatively low. As second step, we adjusted the length of the strings. For this purpose, we conducted a series of FEM simulations for varying string lengths, where we extracted $g_{0,\mathrm{o}}/(2\pi)$ and $g_{0,\mathrm{e}}/(2\pi)$ for the antisymmetric differential in-plane mechanical mode. As described above, the used microwave circuit consists of two parallel mechanically compliant capacitors, each of them with a capacitance of $C_\mathrm{m} = 0.43\,\mathrm{fF}$. The electrical frequency shift per displacement g_{em} is given by the following expression:

$$g_{\mathrm{em}} = -\eta \frac{\omega_\mathrm{e}}{2} \frac{1}{2C_\mathrm{m}} \frac{\partial C}{\partial u} = -\frac{2C_\mathrm{m}}{2C_\mathrm{m} + C_\mathrm{s}} \frac{\omega_\mathrm{e}}{2} \frac{1}{2C_\mathrm{m}} \frac{\partial C}{\partial u}, \tag{34}$$

where u is the mechanical modal amplitude coordinate and $\eta = 2C_\mathrm{m}/(2C_\mathrm{m} + C_\mathrm{s})$ the motional participation ratio. The electromechanical vacuum coupling rate can then be calculated by $g_{0,\mathrm{e}} = 2x_{\mathrm{zpf}} g_{\mathrm{em}}$ with $x_{\mathrm{zpf}} = \sqrt{\hbar/2m_{\mathrm{eff}}\omega_\mathrm{m}}$ as the zero-point amplitude and m_{eff} as the motional mass. FEM simulations revealed values for m_{eff} and x_{zpf} of 1.3 pg and 24.5 fm, respectively. The results for the string length sweep are shown in Supplementary Fig. 1c. Clearly the electromechanical coupling rate increases with the string length while the optomechanical pendant decreases accordingly. For a string length of 9.4 μm the 'zipper' and the string parts of our nanomechanical resonator have roughly the same effective mass and therefore also their zero-point fluctuations are very similar. As a consequence, both the electro- and the optomechanical coupling rate have a decent value which is important for the transducer since conversion requires similar cooperativity for both processes. For this geometry the mechanical mode has a simulated resonance frequency of 10.88 MHz and the coupling rates have simulated values of 60 Hz and 893 kHz.

D. Mechanical oscillator

Since our transducer requires a mechanical motion which modulates maximally the capacitor gap as well as the gap of the optical cavity we included an elastic pinning block which connects the two silicon nanobeams of the 'zipper' at the intersection point with the strings. This pinning block forces the device to feature two in plane differential mechanical modes that create the desired strong electro- and optomechanical interaction and the double clamped tuning fork geometry limits the clamping losses due to elastic wave interference [12]. Another design criterion is the frequency gap between the chosen mechanical mode and other existing resonances of the nanomechanical resonator. The goal here is to avoid hybridization of the chosen mechanical mode with other modes which would cause a significant decrease in the electro- and optomechanical coupling rate. For this purpose we simulated the first mechanical resonances of our structure. The result is shown in Supplementary Fig. 2. There are two in plane differential modes which we distinguish by the terms symmetric and antisymmetric depending on if the strings and the beams of the 'zipper' cavity are oscillating in phase or not. Both of these resonances have significant values for $g_{0,\mathrm{o}}/(2\pi)$ and $g_{0,\mathrm{e}}/(2\pi)$ since the capacitor gap as well as the gap between the nanobeams is strongly modulated. In the end, we chose to work with

the antisymmetric mode since it features slightly bigger coupling rates. Beside these modes there are 3 other family of modes, namely the common in plane, the common out of plane and the differential out of plane modes. However, all of these resonances exhibit negligible electro- and optomechanical coupling. Moreover, all modes are at least 1 MHz away from the mechanical mode we work with. Thus, the other mechanical modes do not influence the performance of the transducer. Also in experiment, we have not seen indications of optomechanical coupling to other modes than to the two differential in plane modes.

E. Device parameter summary

All important device parameters are summarized in Supplementary Table 1. For each parameter the design value from simulation as well as the experimentally extracted value is listed, if available.

Parameter	Simulated value		Measured value	
$\omega_\mathbf{o}/(2\pi)$	193.874	THz	198.081	THz
$\Delta_\mathbf{o}/(2\pi)$	-	-	126	MHz
$\kappa_\mathbf{ex,o}/(2\pi)$	2.15	GHz	0.18	GHz
$\kappa_\mathbf{in,o}/(2\pi)$	0.02	GHz	1.42	GHz
$\omega_\mathbf{e}/(2\pi)$	10.387	GHz	10.497 ($P_\mathrm{o} = 0\,\mathrm{pW}$)<br>10.490 ($P_\mathrm{o} = 92\,\mathrm{pW}$)<br>10.478 ($P_\mathrm{o} = 1556\,\mathrm{pW}$)	MHz
$\Delta_\mathbf{e}/(2\pi)$	-	-	11.84	MHz
$\kappa_\mathbf{ex,e}/(2\pi)$	0.9	MHz	1.15	MHz
$\kappa_\mathbf{in,e}/(2\pi)$	-	-	1.6 ($P_\mathrm{o} = 0\,\mathrm{pW}$)<br>6.1 ($P_\mathrm{o} = 92\,\mathrm{pW}$)<br>13.9 ($P_\mathrm{o} = 1556\,\mathrm{pW}$)	MHz
$L_\mathbf{coil}$	59.8	nH	-	-
$C_\mathbf{s}$	3.0	fF	-	-
$C_\mathbf{m}$	0.9	fF	-	-
$\omega_\mathbf{m}/(2\pi)$	10.9	MHz	11.84	MHz
$\gamma_\mathbf{m}/(2\pi)$	-	-	15 ($P_\mathrm{o} = 0\,\mathrm{pW}$)<br>164 ($P_\mathrm{o} = 92\,\mathrm{pW}$)<br>355 ($P_\mathrm{o} = 1556\,\mathrm{pW}$)	Hz
$x_\mathbf{zpf}$	24.5	fm	-	-
$m_\mathbf{eff}$	1.3	pg	-	-
$g_\mathbf{0,o}/(2\pi)$	893	kHz	662	kHz
$g_\mathbf{0,e}/(2\pi)$	60	Hz	67	Hz

Supplementary Tab. 1. Summary of important device parameters.

Supplementary Note 3. EXPERIMENTAL SETUP

The full measurement setup used for characterizing the microwave-to-optics converter is shown in detail in Supplementary Fig. 3a. It consists of two parts, namely an optical (blue color) and a microwave (red color) reflection setup. The converter is mounted on a stage made out of OFC (oxygen-free copper) attached to the mixing chamber plate of a dilution refrigerator (Bluefors LD250) which is kept at a temperature of $\sim$50 mK, if not specified differently, e.g. in section Supplementary Note 4 B 1 and Supplementary Note 4 B 2.

As light source for our optical setup we use a fiber-coupled tunable external-cavity diode laser (Santec TSL-550 type A) operated around a frequency of $\omega_\mathrm{o}/(2\pi) = 198.0815\,\mathrm{THz}$. Using a 99:1 fiber coupler a small fraction of the light is sent to a wavemeter (λ-meter, Newport WM-1210) for frequency stabilization. The remaining light is divided by a 90:10 fiber coupler into two branches, a low-power signal and a high-power local oscillator arm, required for building an optical heterodyning setup. In the signal arm an acousto-optic modulator (Gooch & Housego T-M200-0.1C2J-3-F2P) is used to shift the light frequency by 200 MHz. Afterwards the light is sent through an single-sideband electro-optic modulator (SSB EOM, Thorlabs LN86S-FC) to generate a single small (approximately 20 dB smaller) optical probe tone detuned by the mechanical frequency $\omega_\mathrm{m}/(2\pi)$. Note that the SSB EOM is operated in such a way that the carrier is not suppressed. Subsequently, the optical signal passes through a variable optical attenuator (VOA, HP8156A) to control the light level that is sent to the sample. Finally, the light is sent to a circulator which routes the light into the dilution refrigerator where a lensed fiber mounted on a stack of attocube® piezo nanopositioners

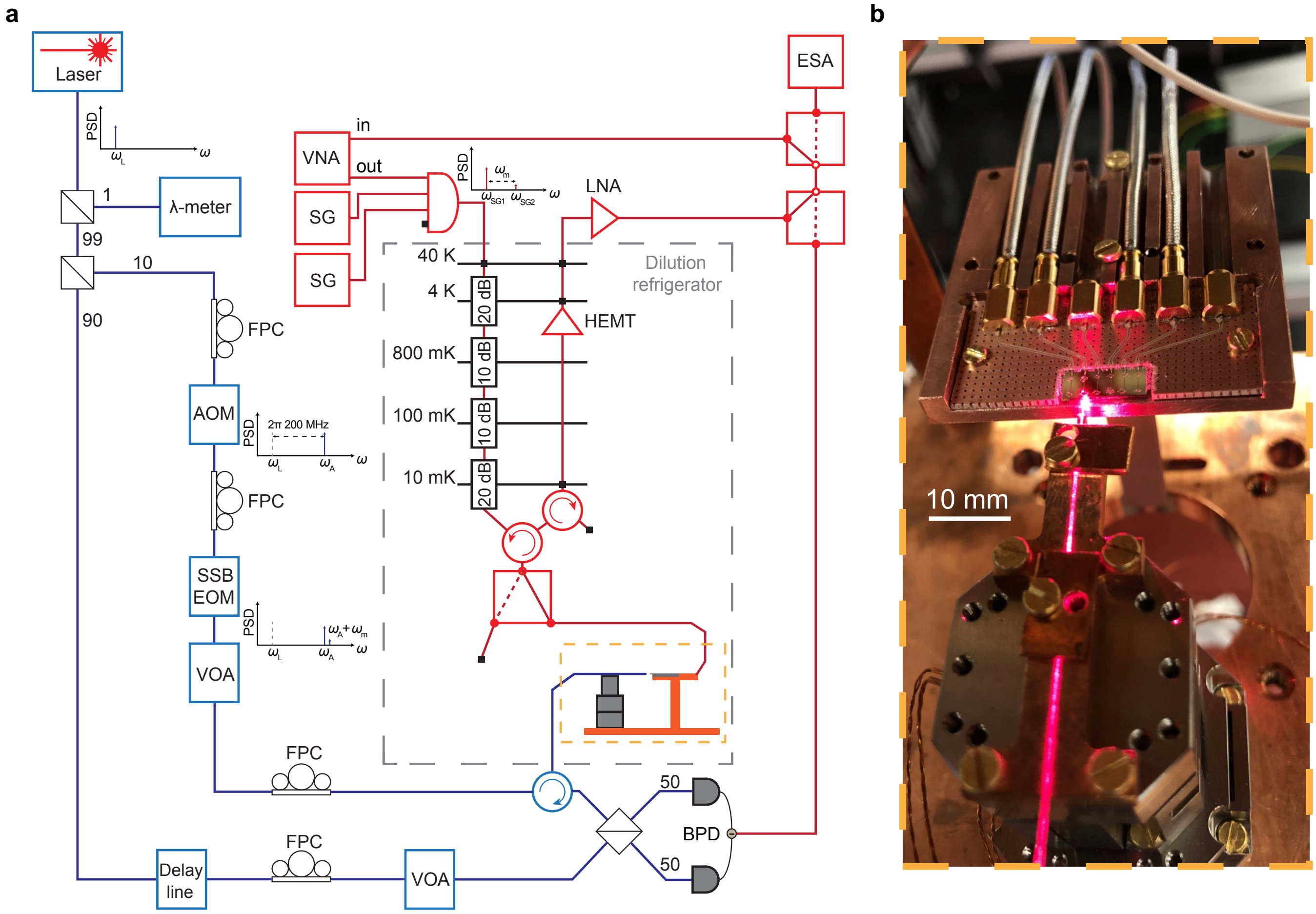

Supplementary Fig. 3. **Optical and microwave conversion measurement setup.** **a**, Schematic of the optical (blue) and microwave (red) setup used for characterizing the microwave-to-optics converter. In the optical setup the laser light is split into two branches, a high-power local oscillator and a low-power signal arm, for building a high-sensitive heterodyning setup. In the signal arm an AOM is used for shifting the light frequency and a SSB EOM is applied for generating a single weak frequency-shifted probe tone. The light reflected from the sample is recombined with the strong local oscillator and measured on a BPD. In the microwave setup the output of the VNA is combined with the output of two SGs and the signals are sent through a cable in the dilution refrigerator to the sample. The microwave signal reflected from the sample is amplified by a HEMT and a LNA. Two microwave switches allow to choose: first if the reflected microwave or the electrical response from the BPD is analyzed and second if the reflected signal is sent to the VNA or the ESA. The spectral position of all signals are indicated schematically at important positions in the setup, i.e. ω_L for the laser frequency and ω_A for the AOM shifted frequency. Acronyms: wavemeter (λ-meter), fiber polarization controller (FPC), acousto-optic modulator (AOM), single-sideband electro-optic modulator (SSB EOM), variable optical attenuator (VOA), balanced photodetector (BPD), vector network analyzer (VNA), microwave signal generator (SG), high-electron-mobility transistor (HEMT), low-noise amplifier (LNA), electronic spectrum analyzer (ESA). **b**, Photograph showing the alignment of the lensed fiber to the chip using the stack of piezo nanopositioners and the mounting of the chip on the printed circuit board in the dilution refrigerator. 650 nm laser light was sent through the optical measurement system instead of telecom wavelengths to achieve visibility of the optical path to the sample.

is used for end-fire coupling to the desired device on the mounted chip with a single-pass coupling efficiency of 64 % (see Supplementary Fig. 3b). The light reflected by the sample is recombined on a 50:50 fiber coupler with the local oscillator signal whose amplitude is kept at roughly 800 μW with an additional variable optical attenuator. Important to note is that the length of both arms were matched to achieve the lowest noise level. The recombined signal is measured eventually on a balanced photodetector (BPD, Thorlabs PDB470C-AC).

In the microwave setup the signals of three devices, i.e. the output port of a vector network analyzer (VNA, Rohde & Schwarz ZNB 20) and two microwave signal sources (SG, Rohde & Schwarz SGS 100A and Rohde & Schwarz SMA 100B), are first combined by a power combiner and then sent together through a cable to the sample in the dilution refrigerator. The signal is attenuated at every temperature stage to eliminate Johnson-Nyquist noise. Using a circulator the microwave signal is routed to the sample which is mounted on a printed circuit featuring coplanar

microwave waveguides to direct the RF signal to the chip (see Supplementary Fig. 3b), or alternatively to a low-temperature 50 Ohm termination by employing a microwave switch mounted also at the mixing chamber plate. On the output side a second circulator is used to isolate the sample from thermal noise coming from the hotter stages above. After this isolator the reflected microwave signal is sent to two amplifiers, i.e. a high-electron-mobility transistor (HEMT, Low-noise factory LNC6-20C) mounted at the 4K-stage in the refrigerator and a low-noise amplifier (LNA, Agile AMT-A0067) positioned outside of the cryostat.

Two microwave switches allow us to decide which signal we want to analyze. The first switch grants us the possibility to choose between the reflected microwave signal or the electronic response of the balanced photodetector. The second switch routes this signal then either to the input port of the VNA or to an electronic spectrum analyzer (ESA, Rohde & Schwarz FSW 26). In conclusion, this allows us to make three types of measurements: 1. measure the microwave resonator with the VNA, 2. spectrally analyze the reflected microwave signal or 3. spectrally analyze the reflected optical signal.

Supplementary Note 4. CHARACTERIZATION

A. Resonator measurements

We characterize the microwave and the optical cavity at a base temperature of 50 mK in our dilution refrigerator by sweeping a weak probe tone in the corresponding frequency range over the used resonances and extract the scattering parameters $|S_{\rm ee}|^2$ (microwave reflection) and $|S_{\rm oo}|^2$ (optical reflection) of the converter. These reflection measurements show a Lorentzian dip around the resonances. The microwave reflection (Supplementary Fig. 4a) has a resonance (with optical pump off) around $\omega_{\rm e}/(2\pi) = 10.5\,{\rm GHz}$ with a total loss rate of $\kappa_{\rm e}/(2\pi) = 2.7\,{\rm MHz}$ and a waveguide coupling rate of $\kappa_{\rm ex,e}/(2\pi) = 1.15\,{\rm MHz}$ leading to a coupling ratio of $\eta_{\rm e} = \kappa_{\rm ex,e}/\kappa_{\rm e} = 0.43$. In contrast, the optical resonance (Supplementary Fig. 4c) at $\omega_{\rm o}/(2\pi) = 198.081\,{\rm THz}$ is much shallower due to the much smaller coupling ratio of $\eta_{\rm o} = 0.11$ connected with a $\kappa_{\rm o}/(2\pi) = 1.60\,{\rm GHz}$ and a $\kappa_{\rm ex,o}/(2\pi) = 0.18\,{\rm GHz}$. The power spectral density of the reflected photons reveals the mechanical resonance frequency at $\omega_{\rm m}/(2\pi) = 11.8424\,{\rm MHz}$ and a mechanical decay rate of $\gamma_{\rm m}/(2\pi) \approx 15\,{\rm Hz}$ (Supplementary Fig. 4b, optical pump off).

B. Calibration

As described in Ref. [13], there is a way to extract the conversion efficiency without the need to know explicitly the gain and attenuation in the measurement setup. In detail, the product of the resonant microwave-to-optics ($P_{\rm oe}(\delta_{\rm e} = 0)$) and optics-to-microwave transduced ($P_{\rm eo}(\delta_{\rm o} = 0)$) power is divided by the product of the off-resonantly measured microwave ($P_{\rm ee}(|\delta_{\rm e}| \gg \kappa_{\rm e})$) and optical reflection power ($P_{\rm oo}(|\delta_{\rm o}| \gg \kappa_{\rm o})$). The square root of this value yields the desired mean bidirectional photon number conversion efficiency, i.e. $|S_{\rm eo}||S_{\rm oe}| = P_{\rm eo}P_{\rm oe}/(P_{\rm ee}P_{\rm oo})$.

However, quantifying the noise quanta added during the conversion requires knowledge of the gain and the attenuation in the microwave as well as the optical measurement. In the following two subsections the calibration procedure for extracting these parameters is explained.

1. Microwave measurement system

Calibrating the microwave measurement system refers to extracting the most important sample parameter on the microwave side, namely the electromechanical coupling rate $g_{0,\rm e}$, the effective microwave gain $\mathcal{G}_{\rm setup,e}$ and the noise added by the chain of amplifiers used to measure the reflected microwave signal $n_{\rm add,setup,e}$ (see section Supplementary Note 3 for details about the setup). This procedure involves a multi-step process consisting of quantifying first $g_{0,\rm e}$, calculating then $n_{\rm add,setup,e}$ and the attenuation on the microwave input side and finally deriving the gain of the chain of amplifiers on the output side.

To extract $g_{0,\rm e}$ we measured the thermal noise power spectrum of the microwave reflection as a function of fridge temperature $T_{\rm fridge}$. Specifically, we swept $T_{\rm fridge}$ from the base temperature of around 7 mK to around 407 mK while recording the noise power spectrum for a weak microwave pump tone so that the electromechanical backaction can be neglected. Each noise measurement was once done for red- and blue-detuning, i.e. $\Delta_{\rm e} = \pm\omega_{\rm m}$, to prove that we are in the low cooperativity limit and to generate two complementary sets of data.

It can be shown that in this limit the noise power spectrum $S_{\rm e}(\omega)$ is described by the following equation (see

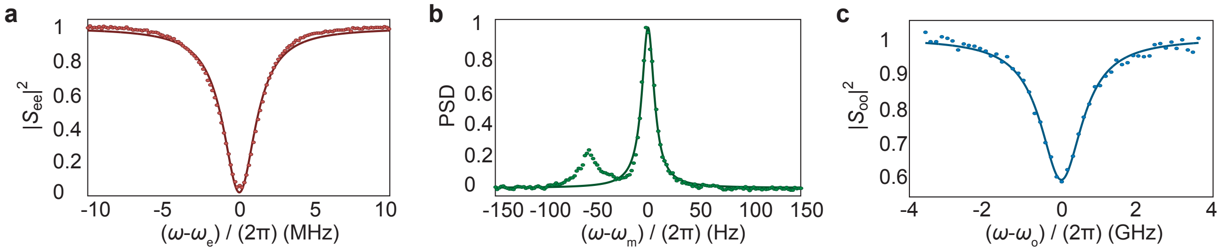

Supplementary Fig. 4. **Cavity and resonator measurements.** **a**, Reflection around the microwave resonance frequency of $\omega_\mathrm{e}/(2\pi) = 10.5\,\mathrm{GHz}$. **b**, Power spectral density of the reflected microwave signal peaking at $\omega_\mathrm{m}/(2\pi) = 11.84\,\mathrm{MHz}$ and **c**, Optical reflection around the cavity resonance of $\omega_\mathrm{o}/(2\pi) = 198.081\,\mathrm{GHz}$. Dots represent the experimental data whereas the lines show the Lorentzian fits.

Ref. [14] for a detailed derivation)

$$\frac{S_\mathrm{e}(\omega)}{P_\mathrm{r}} = \mathcal{O}_\mathrm{e} + \frac{64 n_\mathrm{m}\kappa_\mathrm{ex,e}^2\gamma_\mathrm{m} g_\mathrm{0,e}^2}{(4\Delta_\mathrm{e}^2 + (\kappa_\mathrm{e} - 2\kappa_\mathrm{ex,e})^2)(\kappa_\mathrm{e}^2 + 4(\Delta_\mathrm{e} - \omega)^2)(\gamma_\mathrm{m}^2 + 4(\omega_\mathrm{m} - \omega)^2)}. \tag{35}$$

By normalizing the spectrum $S_\mathrm{e}(\omega)$ by the reflected microwave pump power P_r the dependence on $\mathcal{G}_\mathrm{setup,e}$ of the measurement setup drops out. If we are now able to quantify the mechanical occupation n_m, Eq. (35) will allow us to extract $g_\mathrm{0,e}$ since all other parameters are known from separate measurements.

We have knowledge about n_m, when we know the effective bath temperature $T_\mathrm{m,bath}$ of the mechanical resonator, since they are related to each other by the Bose-Einstein statistics. In the easiest scenario we can set $T_\mathrm{m,bath}$ equal to T_fridge which will be the case if the sample has thermalized with the mixing chamber plate. According to experience this will not be the case for temperatures close to the base temperature of 7 mK but only for elevated temperatures. To verify that the sample is thermalized with the refrigerator we extract $g_\mathrm{0,e}$ for a range of mixing chamber temperatures. When the extracted value for the electromechanical coupling is constant with temperature while assuming that $T_\mathrm{m,bath} = T_\mathrm{fridge}$, the sample is thermalized with its environment and the value of $g_\mathrm{0,e}$ is trustworthy.

Supplementary Fig. 5a shows the extracted values of $g_\mathrm{0,e}/(2\pi)$ for varying fridge temperatures. The value converges for temperatures above 150 mK to a mean value of around 67 Hz. For low temperatures the value varies strongly which is because the assumption $T_\mathrm{m,bath} = T_\mathrm{fridge}$ is not valid anymore. A huge variance in the noise response for low temperatures and in the low cooperativity limit is a well known problem [15] but - as we also observe in experiment - the value stabilizes for stronger pumping or higher temperatures. Here, we only consider the electromechanical coupling rate extracted from the red-detuned measurements because for the blue-detuning $g_\mathrm{0,e}$ was showing consistently a higher value which we attribute to a small amount of parametric gain already having an effect at low cooperativities. This measurement indicates that the sample is only thermalized to the mixing chamber plate for temperatures above 150 mK.

As next step we extract the noise $n_\mathrm{add,setup,e}$ added by the amplifier chain in the microwave output line. For this purpose we examine the background level $\mathcal{O}_\mathrm{e}$ of the thermal noise spectra which is described by the following relation (see again Ref. [14] for a detailed derivation):

$$\mathcal{O}_\mathrm{e} = (1/2 + n_\mathrm{add,setup,e})\frac{4\kappa_\mathrm{ex,e}}{n_\mathrm{d,e}(4\Delta_\mathrm{e}^2 + (\kappa_\mathrm{e} - 2\kappa_\mathrm{ex,e})^2)}. \tag{36}$$

Important to note is that we need to know the microwave intra-cavity photon number $n_\mathrm{d,e}$ to be able to extract $n_\mathrm{add,e}$ which in turn requires knowledge of the exact attenuation $\mathcal{A}_\mathrm{e}$ of the microwave input line in the dilution refrigerator. To gain knowledge of this parameter, we performed independent electromagnetically induced transparency (EIT) spectroscopy measurements which allowed us to extract the term $\sqrt{n_\mathrm{d,e}}g_\mathrm{0,e}$ (measurements are not shown here). The knowledge of $n_\mathrm{d,e}$ as function of microwave pump power provided us with the possibility to calculate $\mathcal{A}_\mathrm{e}$, which equals roughly 76.8 dB in the considered frequency range. This value together with Eq. (36) allowed us to back out the added microwave noise $n_\mathrm{add,e}$ shown in Supplementary Fig. 5b. The average value for temperatures above 150 mK equals 10.4.

Alternatively, the background $\mathcal{O}_\mathrm{e}$ can also be described by the following equation (see Ref. [8] for a detailed derivation)

$$\mathcal{O}_\mathrm{e}(\omega) = \hbar\omega 10^{\mathcal{G}_\mathrm{setup,e}/10}(1/2 + n_\mathrm{add,setup,e}), \tag{37}$$

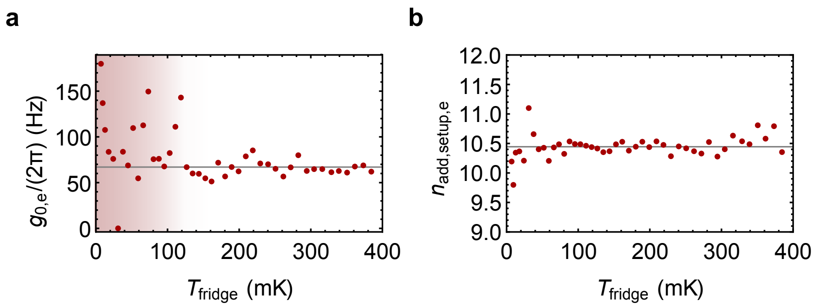

Supplementary Fig. 5. **Microwave calibration.** **a**, Extracted $g_{0,e}/(2\pi)$ for a series of thermal noise spectra with a red-detuned microwave pump tone while sweeping T_{fridge} and inherently assuming that the sample is thermalized to its environment (low pump power limit). The horizontal gray line depicts the average value. At low temperatures the sample is not thermalized to the fridge temperature (red shaded area). **b**, Added noise from the measurement chain to a detected microwave signal extracted from Eq. (36). Also here the horizontal gray line shows the average value.

where $\mathcal{G}_{setup,e}$ is the effective gain of the output side of the microwave setup in dB. Since the only unknown in this equation is $\mathcal{G}_{setup,e}$ we solve it for this parameter which has a value of around 64.1 dB. Thus, we quantified the electromechanical coupling rate and we now know all important parameters of our microwave setup by using this self-consistent calibration method.

2. *Optical measurement system*

The procedure for calibrating the optical measurement system, i.e. determining the effective optical gain $\mathcal{G}_{setup,o}$, the added noise $n_{add,setup,o}$ and from these values the optomechanical coupling rate $g_{0,o}$, is similar from the approach used for the microwave side described above. In essence, we perform a series of thermal noise measurements for varying the refrigerator temperature T_{fridge} and fit these measurements with a numerical model. Eq. (35) cannot be applied because it assumes sideband resolution.

Since our optical detection system is not positioned inside the dilution refrigerator, we are able to measure directly the gain $\mathcal{G}_{setup,o}$ of the optical heterodyning setup. This gain describes the amplification in the process of converting the optical signal coming from the sample to the electrical signal measured on the electrical spectrum analyzer. For the purpose of quantifying $\mathcal{G}_{setup,o}$ we send a well defined amount of optical power in the signal branch and interfere it with a local oscillator power of 800 μW which is the same power as used in all conversion measurements. The combined signal is measured with the balanced photodetector and finally spectrally analyzed by the electronic spectrum analyzer. By dividing the amplitude of the 200 MHz peak (shift frequency of the acousto-optic modulator) of the electric power spectral density by the optical power reflected from the sample we calculate the gain $\mathcal{G}_{setup,o}$ which has a value of around 17.9 dB.

As next step we measure the thermal noise power spectrum on the optics side as a function of refrigerator temperature T_{fridge}. Specifically, we sweep T_{fridge} from a temperature of around 51 mK to around 621 mK while recording the noise power spectrum for a weak optical pump tone, so that the optomechanical backaction can be neglected. The measured electrical power spectral density is converted to the units of number of photons emitted from the optical cavity by dividing by the effective gain $\mathcal{G}_{setup,o}$ and by the optical single photon energy. The corresponding measured power spectral densities are shown in Supplementary Fig. 6a. Three different trends with increasing temperature can be observed in the experimental data. First, the amplitude of the spectrum decreases by more than a factor 2 by going from 51 to 325 mK from whereon it stays rather constant. This maybe unexpected behavior is connected with the second trend, namely that the spectra broaden with increasing T_{fridge}, i.e. the value of γ_m gets larger as reported in the main text. As a consequence, the optomechanical cooperativity decreases which results additionally in a decrease of the amplitude of the spectra. Third, the center frequency of the power spectral density blueshifts with increasing temperature.

The expected number of thermal noise photons emitted by our optical cavity can be described by our numerical model where the electromechanical interaction has been switched off (see section Supplementary Note 1 for details). Assuming now that the sample is thermalized to the mixing chamber plate, we are able to fit the numerical model to the experimental data by using only $g_{0,o}$, which mostly influences the amplitude of the peak, and an offset $\mathcal{O}_o$ as fit parameters while all other parameter values are fixed by independent measurements. The results of these fits are shown in Supplementary Fig. 6a together with the experimental data. It can be seen that the model is able to quantitatively represent all measured thermal noise spectra except for the lowest temperature where the fit expectedly

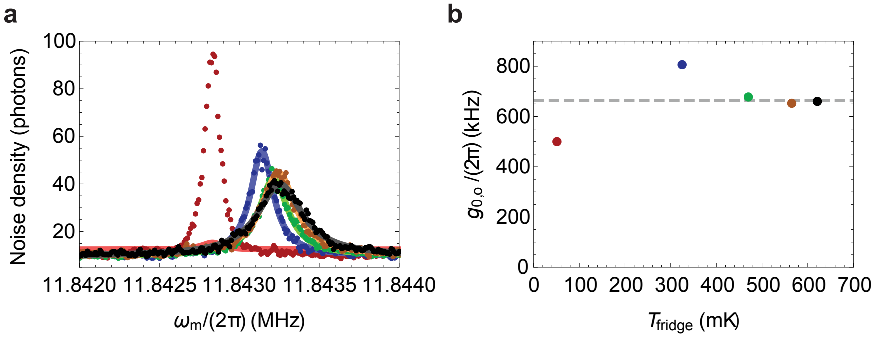

Supplementary Fig. 6. **Extraction of the optomechanical coupling rate $g_{0,o}$.** **a**, Measured (dots) and modeled (lines) thermal noise spectra of the optical reflection in unit of number of photons. The temperature $T_{\rm fridge}$ is swept from 51 to 621 mK in 5 steps: 51 mK (red color), 325 mK (blue color), 469 mK (green color), 565 mK (orange color) and 621 mK (black color). **b**, Values of the optomechanical coupling rate $g_{0,o}$ extracted from this measurement series assuming the sample is thermalized to the mixing chamber plate. The horizontal gray dashed line depicts the average value for the three highest temperatures.

fails. This failure is related to the fact that the sample is not thermalized to the mixing chamber plate for these low temperatures and the assumption $T_{\rm fridge} = T_{\rm m,bath}$ breaks down as discussed in section Supplementary Note 4 B 1. In essence, the model is not able to represent the large amplitude of the noise spectra with a reasonable value of $g_{0,o}$ since the effective bath temperature $T_{\rm m,bath}$ of the mechanics is much larger than assumed. The fitted values for $g_{0,o}/(2\pi)$ as function of $T_{\rm fridge}$ are shown in Supplementary Fig. 6b. For the two lowest temperatures the fit values do not represent the actual optomechanical coupling rate of our device due to thermalization issues and therefore $T_{\rm fridge} \neq T_{\rm m,bath}$. In contrast, the fitted values for higher fridge temperatures are similar. Thus, we take the mean value of these three fits which equals 662 kHz as the actual value for $g_{0,\rm opt}/(2\pi)$. Please note that the minimum temperature at which the sample is thermalized to the fridge is different to Supplementary Fig. 5, because the optical pump leads to absorption heating, as discussed in the main text.

Since the fitted background $\mathcal{O}_{\rm o}$ is already in the units of number of photons we can directly relate it to the number of added noise photons $n_{\rm add,setup,o}$ caused by our imperfect detection

$$\mathcal{O}_{\rm o} = 1/2 + n_{\rm add,setup,o}. \tag{38}$$

Thus, we can calculate the number of added noise photons by subtracting the vacuum noise from the fitted values of $\mathcal{O}_{\rm o}$. This results in an average value of $n_{\rm add,setup,o} = 9.3$.

Alternatively to the approach above, the number of added noise photons can be extracted by modeling our measurement setup as a beam splitter obeying the following input-output formalism

$$a(\omega) = \sqrt{\eta_{\rm qe}}s(\omega) + \sqrt{1-\eta_{\rm qe}}v(\omega), \tag{39}$$

where $a(\omega)$ and $s(\omega)$ are the annihilation operators for the optical field at the balanced photodetector and the sample, respectively, $v(\omega)$ is the annihilation operator for an added thermal noise state and $\eta_{\rm qe}$ represents the quantum efficiency of our measurement. In essence, this equation describes that in the case of a perfect measurement, i.e. $\eta_{\rm qe} = 1.0$, there is no noise added to our output signal, but in all other case there is.

From this equation we can now derive the single sided, symmetrized power spectral density $A_{\rm noise}(\omega)$ as measured by the electronic spectrum analyzer

$$A_{\rm noise}(\omega) = \hbar\omega/2 \int_{-\infty}^{\infty} \left\langle \left(a^\dagger(\omega') + a(\omega')\right)\left(a(\omega') + a^\dagger(\omega')\right)\right\rangle d\omega'. \tag{40}$$

Using this definition and leaving the vacuum field contribution of 1/2 aside, Eq. (39) can be transformed into the following form

$$A_{\rm noise}(\omega) = \eta_{\rm qe}S_{\rm noise}(\omega) + (1-\eta_{\rm qe})V_{\rm noise}(\omega). \tag{41}$$

Here, the quantity $S_{\rm noise}(\omega)$ equals the spectrum emitted directly from the optical cavity, whereas the last term $V_{\rm noise}(\omega)$ describes the power spectral density of the noise that we add to our measurement in addition to the vacuum field contribution of 1/2. By dividing Eq. (41) by $\eta_{\rm qe}$ we can directly see the relation between the quantum efficiency

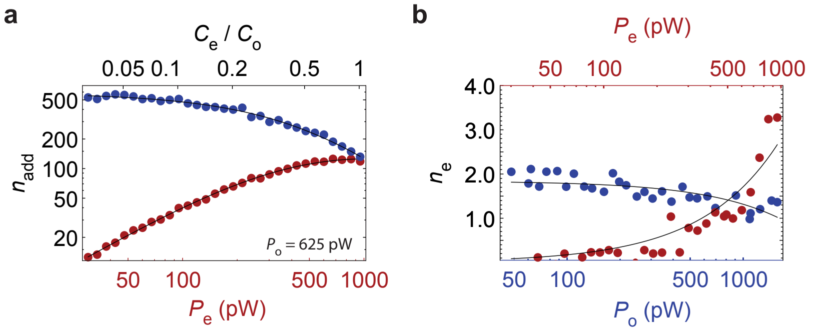

Supplementary Fig. 7. **Transducer output noise and microwave resonator noise a**, Microwave (red, $n_{\mathrm{add,e}}$) and optical (blue, $n_{\mathrm{add,o}}$) added noise photons at the output with respect to microwave pump power and a constant optical pump power of 625 pW (output noise from n_{m} fits to our model as black curves) **b**, Resonator broadband noise with respect to both pumps. The microwave resonator noise n_{e} increases linearly with microwave pump power ($P_{\mathrm{o}} = 625\,\mathrm{pW}$, black line is a fit to $2.8 \times 10^{-3} P_{\mathrm{e}}$). In contrast, we attribute the slight decrease with increasing optical pump power ($P_{\mathrm{e}} = 601\,\mathrm{pW}$, fit to $1.8 - 5.2 \times 10^{-4} P_{\mathrm{o}}$ as black line) to the effect of optical heating on the microwave resonator (see Fig. 2e in the main text).

of the measurement and the added noise photons

$$\frac{A_{\mathrm{noise}}(\omega)}{\eta_{\mathrm{qe}}} = S_{\mathrm{noise}}(\omega) + \frac{1-\eta_{\mathrm{qe}}}{\eta_{\mathrm{qe}}} V_{\mathrm{noise}}(\omega). \tag{42}$$

In this equation the quantities $A_{\mathrm{noise}}(\omega)/\eta_{\mathrm{qe}}$ and $S_{\mathrm{noise}}(\omega)$ represent noise spectra with the background observed in the experiment and a background coming from vacuum noise, respectively. Consequently, the last term equals directly the added noise photon number $n_{\mathrm{add,setup,o}}$.

To extract $n_{\mathrm{add,setup,o}}$ we first have to find the efficiency of our measurement. For that we measure the noise output at elevated fridge temperatures to guarantee good thermalization to the sample. Provided a known mechanical occupation, we then fit the proportionality η_{qe} between the uncalibrated noise peaks observed at the detector (normalized to its noise floor) and the expected signal in units of photons at the converter output from the theoretical model without further parameters. This results in $\eta_{\mathrm{qe}} = 0.099$. Inserting this number into the last term of Eq. (42) yields an added system noise photon number $n_{\mathrm{add,setup,o}} \approx 9.1$, in good agreement with the previous calibration method using the measured effective gain of our heterodyning setup (Supplementary Fig. 6).

C. Conversion noise and heating

1. *Microwave pump*

Analogue to Fig. 4d in the main text, the microwave and optical output noise of the transducer can be plotted with respect to electrical pump power (see Supplementary Fig. 7a). For a fixed optical pump power ($P_{\mathrm{o}} = 625\,\mathrm{pW}$), the output noise of the microwave resonator $n_{\mathrm{add,e}}$ increases with electrical pump power because of an increasing photon-phonon coupling rate Γ_{e}. In turn, the optical output noise $n_{\mathrm{add,o}}$ decreases with increasing electrical pump powers because of an increasing Γ_{e}, until microwave and optical output noise intersect for matching cooperativities, as can also be seen in Fig. 4d of the main text. As shortly discussed there, this proves that optical and mechanical output share the same bath. As long as the mechanical noise is the main contribution, the output noise is mainly determined by Eq. (13d). The absolute square of coefficient $\alpha_{j,\mathrm{m}}$ describes the relation between mechanical occupation and photon noise and is proportional to the individual cooperativities. Thus, if on the one hand $C_{\mathrm{e}} \approx C_{\mathrm{o}}$ respectively $4G_{\mathrm{e}}^2/\kappa_{\mathrm{e}} \approx 4G_{\mathrm{o}}^2/\kappa_{\mathrm{o}}$, and on the other hand $n_{\mathrm{add,e}} \approx n_{\mathrm{add,o}}$, the mechanical occupation n_{m} must be equal for optomechanical and electromechanical resonator.

2. *Microwave resonator*

Apart from the optics-related boost of mechanical noise and thereby noise photons added in the frequency-range of the transducer bandwidth, we could also observe a minor contribution from broadband resonator noise n_{e} (see Supplementary Fig. 7b). It linearly increases with electrical power. The small decrease we observe with increasing optical power might be explained by effects of the optical pump on the microwave resonator.

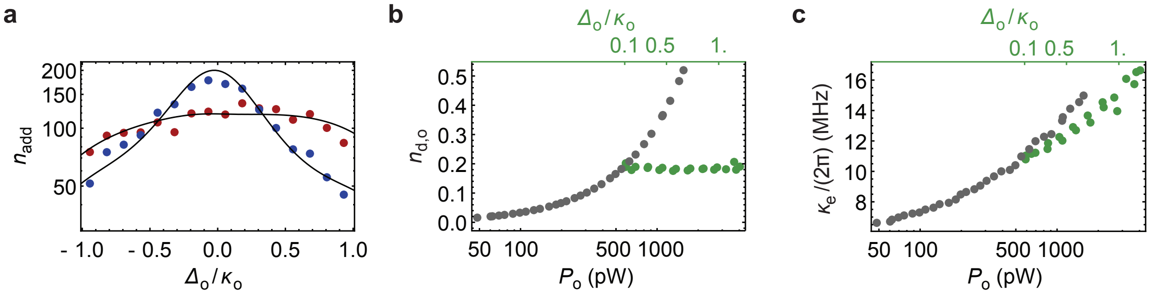

Supplementary Fig. 8. **Transducer parameters vs detuning.** **a**, Electrical (red) and optical (blue) added noise at the transducer output with respect to the optical detuning ratio Δ_o/κ_o. **b**, Drive photons in the optical cavity $n_{d,o}$ vs. optical pump power in the waveguide P_o for constant detuning $\Delta_o/\kappa_o \approx 0.09$ (gray) and varying detuning with $n_{d,o} \approx 0.19$ (green). **c**, Microwave resonator linewidth $\kappa_e/(2\pi)$ with respect to optical pump power. The resonator linewidth increase is stronger when the optical pump is applied near resonance (gray), compared to the detuning dependent case with lower and constant intra-cavity photon number $n_{d,o}$ (green).

3. Detuning dependencies

The optomechanical gain [16, 17] strongly depends on the pump detuning. Figure 3 in the main text shows the dependency of the total transduction ζ (Eq. (15)), the pure conversion efficiency θ (Eq. (16)), and the gain $\mathcal{G}$ (Eq. (17)) of the transducer ($\omega \approx \omega'_m$) on the optical detuning for a constant optical intra-cavity photon number of $n_{d,o} = 0.185 \pm 0.015$ and thereby a constant optomechanical coupling rate $G_o = g_{0,o}\sqrt{n_{d,o}}$. As described in the main text, the gain increases rapidly for vanishing detuning $\Delta_o \to 0$, while the conversion approaches 0. The corresponding numbers of the noise photons added to the electrical (red) and optical output (blue) are given in Supplementary Fig. 8. The electrical output noise is flattened compared to the optical output noise. The reason can be found in the phonon-photon coupling coefficient Eq. (13d) because thermal mechanical occupation is the main noise source. The coefficient for coupling between phonons and the microwave (optical) photon output is proportional to the product of $G_e\chi_e$ ($G_o\chi_o$). While $G_e\chi_e$ is rather constant apart from changes in κ_e due to optical heating and G_o was kept constant vs. optical detunings by varying the pump power, χ_o and thereby $n_{add,o}$ depend strongly on the optical detuning and the optical output noise shows a peak around $\Delta_o = \omega_m$ ($\Delta_o/\kappa_o \approx 0.07$).

In conjunction with the optical power sweep for constant optical detuning $\Delta_o/(2\pi) \approx 126\,\mathrm{MHz}$ presented in the main text in Fig. 4c, it is then possible to compare heating rates for the same optical pump powers P_o (optical powers in the waveguide) but different intra-cavity photon numbers $n_{d,o}$ (circulating power in the cavity) (Supplementary Fig. 8b). The deterioration of the microwave resonator linewidth κ_e is a good indicator for optical heating. As can be seen in Supplementary Fig. 8c, κ_e rises faster with increasing optical pump power P_o when the optical detuning is fixed and the intra-cavity photon number also increases (gray) compared to the case where the intra-cavity photon number is kept constant by varying Δ_o (green). At a pump power $P_o \approx 500\,\mathrm{pW}$ and $\Delta_o/\kappa_o \approx 0.09$ we find a consistent $\kappa_e/(2\pi)$ of 11.1 MHz for both measurement sweeps. If P_o is increased to 1500 pW, $\kappa_e/(2\pi)$ was broadened to 14.8 MHz (33 % increase) for constant detuning $\Delta_o/\kappa_o \approx 0.09$ ($n_{d,o} \approx 0.5$), but only to 12.8 MHz (15 % increase) for a different detuning $\Delta_o/\kappa_o \approx 0.65$ but a constant intra-cavity photon number $n_{d,o} \approx 0.19$. However, the fact that κ_e increases also in the latter case of constant $n_{d,o}$ reveals that the intra-cavity photon number is not the only deciding factor for the heating rate. Similar trends can be found for the mechanical occupation. For $\Delta_o/\kappa_o \approx 0.09$ and $P_o = 500\,\mathrm{pW}$ ($n_{d,o} \approx 0.19$), we find again a consistent mechanical bath temperature $T_{m,bath}$ of 0.65 K for both measurement runs. If the pump power is increased to $P_o = 1500\,\mathrm{pW}$, $T_{m,bath}$ rises by 23 % to 0.85 K ($n_{d,o} \approx 0.5$, $\Delta_o/\kappa_o \approx 0.09$), while it only heats up to 0.70 K or 8 %, if the intra-cavity photon number is kept at 0.19 ($\Delta_o/\kappa_o \approx 0.65$). We attribute the higher absorption heating by actual intra-cavity photons to the fact that the photonic crystal cavity has a direct physical connection to the microwave circuit by the mechanically compliant capacitors. If the optical photons do not enter the cavity but remain in the waveguide, there is only an indirect connection to the circuit via the silicon membrane and stray light.

D. Bidirectionality and calibration uncertainties

Similar to Fig. 2a in the main text, the total spectrum can also be evaluated at an optical detuning of $\Delta_o \approx \kappa_o/2$, where the optomechanical gain has its minimum, as shown in Fig. 3 of the main text. In case of optics-to-microwave conversion, a coherent signal applied to the upper optical sideband gets partly reflected (Supplementary Fig. 9a) and

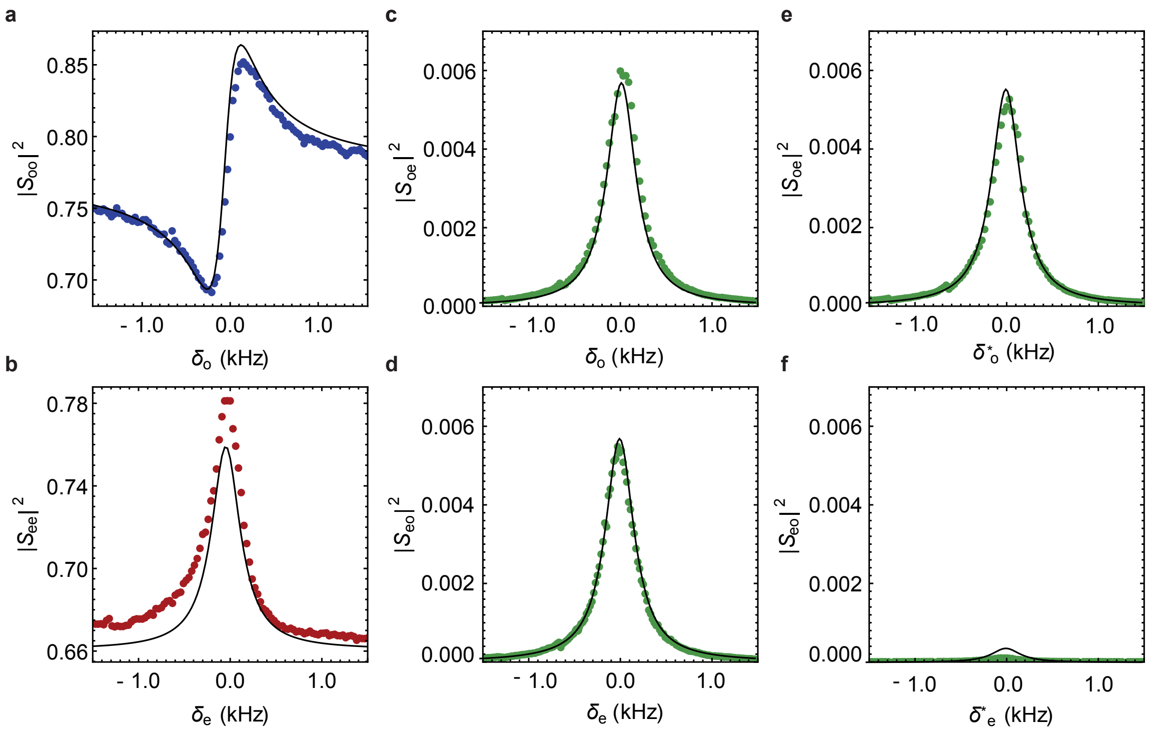

Supplementary Fig. 9. **Measured scattering parameter spectra.** Measured reflected and transmitted spectra (dots) with theory (lines) around the upper and lower mechanical sideband, $\delta_j = \omega - (\omega_{\mathrm{d},j} - \omega_{\mathrm{m}})$ and $\delta_j^* = \omega - (\omega_{\mathrm{d},j} + \omega_{\mathrm{m}})$ respectively, at an optical pump detuning of $\Delta_{\mathrm{o}} = \kappa_{\mathrm{o}}/2$, where the transducer gain is minimal and the total (internal) pure conversion efficiency reaches 0.017 % (1.6 %). **a**, Probe reflection from the optical cavity. **b**, Probe reflection from the microwave resonator. **c**, Upper sideband of microwave-to-optics transduction. **d**, Upper sideband of optics-to-microwave transduction. **e**, Lower sideband of microwave-to-optics transduction. **f**, Lower sideband of optics-to-microwave transduction.

partly converted to the microwave port, where the converted photons exit the transducer predominantly at the upper sideband (Supplementary Fig. 9d vs. f). The lower sideband suppression ratio is given by $\chi_{\mathrm{e}}^*/\chi_{\mathrm{e}} >> 1$. In the reverse direction, the applied microwave signal gets almost symmetrically transduced to the optical upper and lower sideband ($\chi_{\mathrm{o}}^*/\chi_{\mathrm{o}} \approx 1$), as can be seen in Supplementary Fig. 9c and e. As mentioned in the main text, we define the efficiency of the transducer only between the upper optical and upper microwave sideband and neglect any power converted to the optical or microwave lower sideband. With this definition, the transducer is symmetric in both directions, both according to theory and experimentally as shown in Supplementary Fig. 9c and d.

When the two directions are determined separately, uncertainties in the independent calibration of the system gains and losses can lead to differences in the calculated efficiencies in the two directions. Close to zero optical detuning we have experimentally seen deviations by +30% (-35%) of the calculated optics to microwave (microwave to optics) directions. Even though we cannot fully rule out the possibility of unknown or higher order effects that are not taken into account, we attribute this imbalance not to an actual symmetry-breaking in the system, but to calibration difficulties associated with changes of the reference baseline or possible retroflections due to three main reasons: i) Changes in the optical pump power or detuning alter the resonance frequency of the microwave resonator. As a consequence, frequency-dependent changes in the microwave baseline have to be explicitly taken into account. ii) The optical pump has not only an influence on the characteristics of the microwave resonator but, to a certain extend, also the baseline itself. iii) Our resonators are highly under-coupled and therefore comparably sensitive to changes in the background. As an example to demonstrate the sensitivity of our extracted parameters, the offset observed between the theoretical prediction and the measured data in Supplementary Fig. 9b is equivalent to an uncertainty in the baseline difference between off-resonant and on-resonant signal frequency of $< 0.1\,\mathrm{dB}$.

A quantity that is insensitive to imperfect measurements of the individual gains and losses is the mean bidirectional transduction $|S_{\mathrm{eo}}||S_{\mathrm{oe}}|$ [13] as well as the line shape of the two reflection coefficients $|S_{\mathrm{ee}}|$ and $|S_{\mathrm{oo}}|$ which both agree well with theory and the main transduction, as shown in Fig. 2a of the main text. The reported efficiencies therefore refer to this method as it requires the least amount of knowledge about the measurement system.

Supplementary Note 5. DECOMPOSITION OF THE TRANSDUCER PERFORMANCE

To get a better understanding of the influence of the various processes involved in the transduction process it can be instructive to decompose Eq. (15) into a product of factors that describe the separate steps independently. Firstly, the complete transduction can be separated in a term for the pure conversion (Eq. (16)) and two terms for the electro- and optomechanical gain (Eqs. (17)), as discussed above. While also the factors responsible for the cavity to waveguide coupling efficiency η_e and η_o can be easily extracted, it is not so straightforwardly possible to separate the remaining part into the product of two factors only describing the electro- and optomechanical conversion efficiency (η_em and η_om). From a physical point of view this can be understood by the fact that these processes are actually not independent but influence each other, i.e. the electromechanical coupling rate acts as an additional damping term for the optomechanical conversion and vice versa.

To be able to extract values for η_em and η_om we approximate Eq. (16) in the vicinity of the mechanical resonance (i.e. $\omega \approx \omega_\mathrm{m}$) by

$$\theta \approx \theta_\mathrm{app} = \eta_\mathrm{e}\eta_\mathrm{o}\Gamma_\mathrm{o}\frac{4\Gamma_\mathrm{e}}{(\gamma_\mathrm{m}+\Gamma_\mathrm{e})^2}, \tag{43}$$

which is valid for small optomechanical damping rates as is the case for our non-sideband resolved optical cavity. By defining η_om as the ratio of Γ_o to the mechanical decay rate γ_m, we can separate this equation into the desired form

$$\theta_\mathrm{app} = \eta_\mathrm{e}\eta_\mathrm{o}\frac{\Gamma_\mathrm{o}}{\gamma_\mathrm{m}}\frac{4\Gamma_\mathrm{e}\gamma_\mathrm{m}}{(\gamma_\mathrm{m}+\Gamma_\mathrm{e})^2} = \eta_\mathrm{e}\eta_\mathrm{o}\eta_\mathrm{om}\eta_\mathrm{em} \tag{44}$$

with $\eta_\mathrm{om} = \Gamma_\mathrm{o}/\gamma_\mathrm{m}$ and $\eta_\mathrm{em} = 4\Gamma_\mathrm{e}\gamma_\mathrm{m}/(\gamma_\mathrm{m}+\Gamma_\mathrm{e})^2$. This derivation is analogous to the approach in Ref. [18] and relying on an approximation that is not valid in the limit of large electro- and optomechanical damping rates. As a result the optomechanical conversion efficiency is systematically over- and the electromechanical one underestimated.

Nevertheless, the factorization in Eq. (44) works reasonable well in the parameter range where our transducer operates. This allows to separate the influence of the various sub-steps of the conversion process on the total transduction efficiency. In Supplementary Table 2 we report the extracted efficiencies for the measurement shown in Fig. 2a in the main text. It can be clearly seen that the value for the total pure conversion efficiency $\theta_\mathrm{app} = \eta_\mathrm{e}\eta_\mathrm{o}\theta_\mathrm{int,app}$ is limited by three factors, namely the two waveguide coupling efficiencies η_j and the pure optomechanical conversion efficiency η_om, which originates from the poor sideband resolution of the optical cavity. In contrast, for the total transduction $\zeta_\mathrm{app} = \eta_\mathrm{e}\eta_\mathrm{o}\zeta_\mathrm{int,app}$ only the small η_j prevent a close to 100 % total transduction efficiency.

Due to the fact that the mechanical as well as the microwave resonator degrade with optical pump power P_o, it is instructive to examine how this influences the efficiency of the various processes, as illustrated in Supplementary Fig. 10. While the optomechanical conversion efficiency η_om increases with increasing P_o, η_e and η_em are decreasing. However, since for low pump powers the η_om is increasing faster than the product of η_e and η_em is degrading, the total transduction ζ is gaining in amplitude here and as shown in Fig. 2c of the main text. For larger optical pump powers the microwave and mechanical quality factor reduction becomes too severe and the total transduction is decreasing again with a maximum around $P_\mathrm{o} \approx 1000\,\mathrm{pW}$.

Parameter	Variable	Extracted value
total transduction efficiency	ζ	0.0107
microwave waveguide coupling efficiency	η_e	0.0988
optical waveguide coupling efficiency	η_o	0.1125
internal transduction efficiency	$\zeta_\mathrm{int} = \zeta/(\eta_\mathrm{e}\eta_\mathrm{o})$	0.9589
pure electromechanical conversion efficiency	$\eta_\mathrm{em} = 4\Gamma_\mathrm{e}\gamma_\mathrm{m}/(\gamma_\mathrm{m}+\Gamma_\mathrm{e})^2$	0.9090
electromechanical gain	$\mathcal{G}_\mathrm{e}$	1.0603
electromechanical transduction efficiency	$\zeta_\mathrm{em} = \mathcal{G}_\mathrm{e}\cdot\eta_\mathrm{em}$	0.9639
pure optomechanical conversion efficiency	$\eta_\mathrm{om} = \Gamma_\mathrm{om}/\gamma_\mathrm{m}$	0.0091
optomechanical gain	$\mathcal{G}_\mathrm{o}$	110.40
optomechanical transduction efficiency	$\zeta_\mathrm{om} = \mathcal{G}_\mathrm{o}\cdot\eta_\mathrm{om}$	1.0072
approximated internal pure conversion efficiency	$\theta_\mathrm{int,app} = \eta_\mathrm{em}\cdot\eta_\mathrm{om}$	0.0083
approximated internal transduction efficiency	$\zeta_\mathrm{int,app} = \mathcal{G}_\mathrm{e}\cdot\mathcal{G}_\mathrm{o}\cdot\theta_\mathrm{int,app}$	0.9709

Supplementary Tab. 2. Summary of the efficiencies of all sub-steps important in the transduction process, extracted from the measurement shown in Fig. 2a of the main text and evaluated at the mechanical resonance frequency (i.e. $\delta_j = 0$).

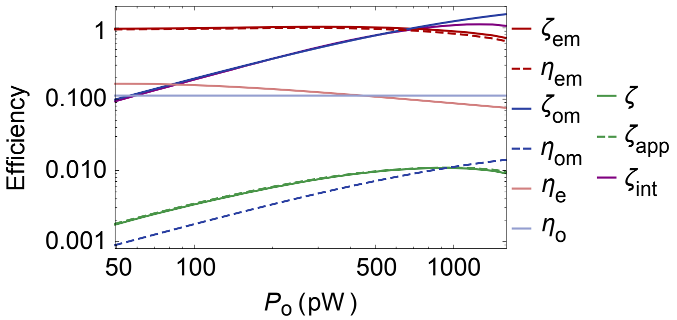

Supplementary Fig. 10. **Conversion efficiencies as a function of P_o.** The directly measured efficiencies ζ, η_e, η_o, $\zeta_{int} = \zeta/(\eta_e\eta_o)$ and the inferred efficiencies η_{em}, η_{om}, ζ_{em}, ζ_{om} and ζ_{app} of all sub-steps involved in a full transduction process as function of the optical pump power P_o. The shown values are extracted using the data shown in Fig. 2c of the main text.

Supplementary Note 6. PERFORMANCE AS A CLASSICAL PHASE MODULATOR

Considering our device as a classical phase modulator the most important figure of merit is its value for V_π. This value represents the voltage amplitude of an input microwave signal that generates a π phase shift of the optical output with respect to its input. In this section we derive the equation for calculating V_π for a triple-resonant electro-opto-mechanical system. Moreover, we also estimate the required energy E_{bit} to encode a classical bit on an optical carrier signal. Finally, we compare the values of both figures of merit to the literature.

A. Modulation voltage V_π

Starting point for the derivation of V_π is the relation between the optomechanical coupling rate $g_{0,o}$ and the phase shift $\Delta\phi$ experienced by an optical photon due to the number of phonons n_{ph} occupying the mechanical resonator [19]

$$g_{0,o}\sqrt{n_{ph}} = \frac{\Delta\phi}{\tau}, \tag{45}$$

where τ is the lifetime of the photons in the optical cavity for which holds $\tau = 1/\kappa_o$. The number of phonons that are generated by a microwave signal P_e at the microwave resonance frequency ω_e is given by [18, 20]

$$n_{ph} = |\mathbf{\Theta}_{3,1}(\omega)|^2 \frac{P_e}{\hbar\omega_e}, \tag{46}$$

where $\mathbf{\Theta}$ is the matrix defined by $[-i\omega\mathbf{I} - \mathbf{A}]^{-1}.\mathbf{B}$ using the definitions in section Supplementary Note 1 and its element $\mathbf{\Theta}_{3,1}(\omega)$ is given by

$$\mathbf{\Theta}_{3,1}(\omega) = -\frac{i\sqrt{\kappa_e\eta_e}\,G_e\chi_e\chi_m}{1 + [\chi_m - \chi_m^*]\left[G_e^2(\chi_e - \chi_e^*) + G_o^2(\chi_o - \chi_o^*)\right]}, \tag{47}$$

with the susceptibilities $\chi_j(\omega)$ and $\chi_j^*(\omega)$ defined above. Finally, the microwave input power P_e can be related to a sinusoidal peak voltage V_p by

$$P_e = \frac{V_p^2}{2Z_e}, \tag{48}$$

where Z_e is the impedance of the microwave input waveguide, in our case $50\,\Omega$. Combining the Eqs. (46) to (48) and solving for the voltage V_π that causes a $\Delta\phi$ of value π leads to

$$V_\pi(\omega) = \frac{1}{|\mathbf{\Theta}_{3,1}(\omega)|}\frac{\pi\kappa_o}{g_{0,o}}\sqrt{\hbar\omega_e 2Z_e}. \tag{49}$$

The value for V_π is minimal at the mechanical resonance (i.e. $\omega = \omega_m$) since the conversion of microwave photons to phonons given by $|\mathbf{\Theta}_{3,1}(\omega)|$ is maximum.

There are two important scenarios we want to consider here where the equations can be significantly simplified. The first scenario is the case of perfectly red-detuned pump tones (i.e. $\Delta_\mathrm{e} = \Delta_\mathrm{o} = \omega_\mathrm{m}$) and sideband resolution on the microwave as well as on the optical side (i.e. $\kappa_\mathrm{e}, \kappa_\mathrm{o} \ll \omega_\mathrm{m}$) so that we can use here the simplified expression $\Gamma_j = 4G_j^2/\kappa_j = \mathcal{C}_j\gamma_\mathrm{m}$ for the electro- and optomechanical damping rates Γ_j with cooperativities $\mathcal{C}_j$. Then Eq. (47) can be approximated by

$$\mathbf{\Theta}_{3,1}(\omega_\mathrm{m}) \approx -i2\sqrt{\eta_\mathrm{e}}\sqrt{\frac{\Gamma_\mathrm{e}}{(\gamma_\mathrm{m}+\Gamma_\mathrm{e}+\Gamma_\mathrm{o})^2}} = -i2\sqrt{\frac{\eta_\mathrm{e}}{\gamma_\mathrm{m}}}\sqrt{\frac{\mathcal{C}_\mathrm{e}}{(1+\mathcal{C}_\mathrm{e}+\mathcal{C}_\mathrm{o})^2}}. \tag{50}$$

This expression shows that the number of phonons generated due to a microwave signal is maximum when the conditions $\Gamma_\mathrm{e} = \gamma_\mathrm{m}$ and $\Gamma_\mathrm{o} \ll \gamma_\mathrm{m}$ are fulfilled. This will result then in rate matching between the electromechanical damping rate Γ_e and the effective loss rate of the mechanical resonator γ_m leading to the best phonon conversion [21]. For unmatched rates, a larger part of the microwave photons will be reflected and not converted to phonons. The second condition of having a vanishing Γ_o comes from the fact that the conversion of phonons to optical photons leads to an additional loss channel of the mechanical resonator limiting therefore the achievable value for n_ph and $\Delta\phi$ for a given microwave signal. This shows the difference to the operation mode of a microwave-to-optics converter where the condition $\Gamma_\mathrm{e} = \Gamma_\mathrm{o} \gg 1$ has to be fulfilled for efficient conversion. Consequently the value for V_π can be calculated by

$$V_\pi(\omega_\mathrm{m}) \approx \frac{1}{2}\sqrt{\frac{1}{\eta_\mathrm{e}}}\sqrt{\frac{(\gamma_\mathrm{m}+\Gamma_\mathrm{m}+\Gamma_\mathrm{o})^2}{\Gamma_\mathrm{e}}}\frac{\pi\kappa_\mathrm{o}}{g_{0,\mathrm{o}}}\sqrt{\hbar\omega_\mathrm{e}2Z_\mathrm{e}} = \frac{1}{2}\sqrt{\frac{\gamma_\mathrm{m}}{\eta_\mathrm{e}}}\sqrt{\frac{(1+\mathcal{C}_\mathrm{e}+\mathcal{C}_\mathrm{o})^2}{\mathcal{C}_\mathrm{e}}}\frac{\pi\kappa_\mathrm{o}}{g_{0,\mathrm{o}}}\sqrt{\hbar\omega_\mathrm{e}2Z_\mathrm{e}}. \tag{51}$$

The second scenario describes our transducer. Here, we do not achieve sideband resolution on the optical side but are in the limit of $\kappa_\mathrm{e} \ll \omega_\mathrm{m}$ and $\kappa_\mathrm{o} \gg \omega_\mathrm{m}$ leading to $\Gamma_\mathrm{e} = \mathcal{C}_\mathrm{e}\gamma_\mathrm{m}$ and $\Gamma_\mathrm{o} \to 0$. For Eq. (47) holds then

$$\mathbf{\Theta}_{3,1}(\omega_\mathrm{m}) \approx -i2\sqrt{\eta_\mathrm{e}}\sqrt{\frac{\Gamma_\mathrm{e}}{(\gamma_\mathrm{m}+\Gamma_\mathrm{e})^2}} = -i2\sqrt{\frac{\eta_\mathrm{e}}{\gamma_\mathrm{m}}}\sqrt{\frac{\mathcal{C}_\mathrm{e}}{(1+\mathcal{C}_\mathrm{e})^2}}. \tag{52}$$

The maximum for this expression is given again for a rate matching of Γ_e and γ_m (neglecting any power dependency of η_e and γ_m). Important to note is also that this approximation for $\mathbf{\Theta}_{3,1}$ is valid as long as the optical detuning is smaller than the optical linewidth, i.e. $\Delta_\mathrm{o} \ll \kappa_\mathrm{o}$ and not only at the resonance condition $\Delta_\mathrm{o} = \omega_\mathrm{m}$. In this scenario the voltage V_π can be calculated by

$$V_\pi(\omega_\mathrm{m}) \approx \frac{1}{2}\sqrt{\frac{1}{\eta_\mathrm{e}}}\sqrt{\frac{(\gamma_\mathrm{m}+\Gamma_\mathrm{e})^2}{\Gamma_\mathrm{e}}}\frac{\pi\kappa_\mathrm{o}}{g_{0,\mathrm{o}}}\sqrt{\hbar\omega_\mathrm{e}2Z_\mathrm{e}} = \frac{1}{2}\sqrt{\frac{\gamma_\mathrm{m}}{\eta_\mathrm{e}}}\sqrt{\frac{(1+\mathcal{C}_\mathrm{e})^2}{\mathcal{C}_\mathrm{e}}}\frac{\pi\kappa_\mathrm{o}}{g_{0,\mathrm{o}}}\sqrt{\hbar\omega_\mathrm{e}2Z_\mathrm{e}}. \tag{53}$$

Using the set of parameters of our transducer we calculate a minimum value of V_π of around $16\,\mu$V which we reach for low optical pump power (i.e. $P_\mathrm{o} = 92\,$pW) and a microwave pump power of $P_\mathrm{e} = 409\,$pW. Higher optical pump powers lead to an increase of V_π due to the discussed optical heating effects leading to an increasing γ_m and a decreasing η_e.

B. Estimate for the energy-per-bit E_bit

Another important figure of merit related to the device efficiency is the microwave energy E_bit required to encode a bit on an optical carrier signal where we assume again the case of phase modulation. As a rough estimate we calculate this value as the ratio of the microwave signal power $P_\pi = V_\pi^2/(2Z_\mathrm{e})$ required to achieve a π optical phase shift to the effective bandwidth Γ_conv. As discussed above V_π is minimal for $\Gamma_\mathrm{e} = \gamma_\mathrm{m}$ which results in a modulation bandwidth of $2\gamma_\mathrm{m}$. When we work at this point for a low power optical signal (i.e. $P_\mathrm{o} = 92\,$pW) our device requires an energy E_bit of around $1.3\,$fJ. Note that in this discussion we have neglected the microwave pump power P_e that is required to achieve the parametric enhancement of the electromechanical coupling rate $G_\mathrm{e} = \sqrt{n_\mathrm{d,e}}g_{0,\mathrm{e}}$ which equals a value of around $409\,$pW.

C. Comparison of the values of V_π and E_bit to literature

Various approaches can be used to implement an optical phase modulator ranging from electro-optomechanics [13, 22, 23] over piezo-optomechanics [24–27] to electro-optics [20, 28, 29]. Our minimum value for V_π represents a

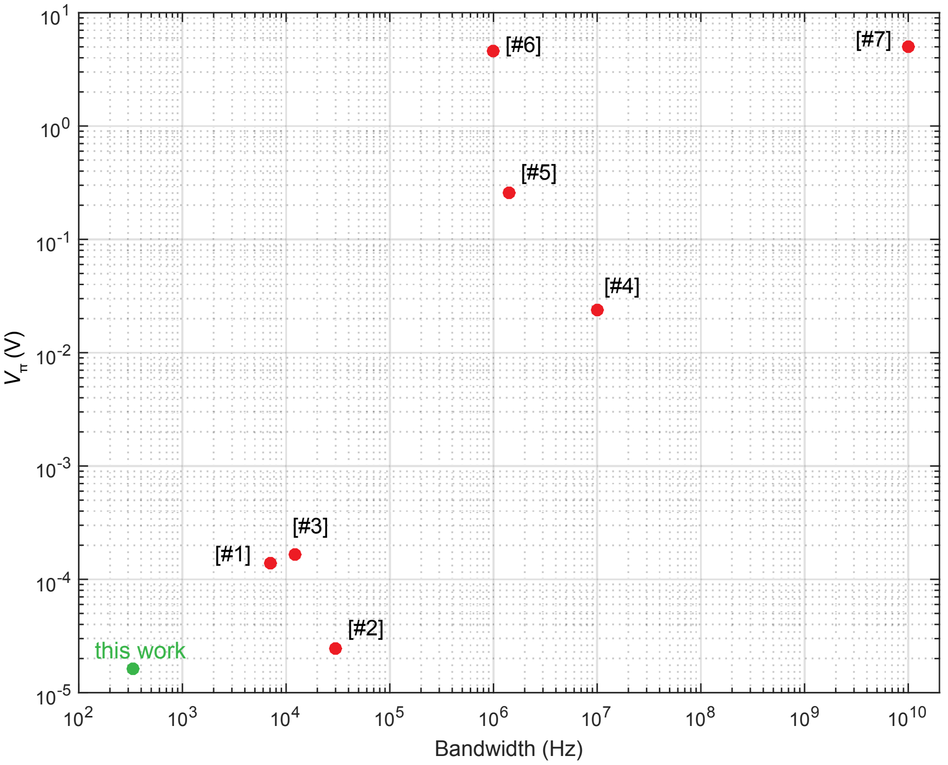

Supplementary Fig. 11. **Performance comparison of phase modulator implementations.** Values of voltage V_π and bandwidth of various optical phase modulator platforms. Our implementation offers a new record value of V_π which is more than 2×10^5 times better than a commercial Thorlabs electro-optic modulator LN53S-FC (#7). The price we pay for the very efficient modulation of our triple-resonant system is the low bandwidth. Other reported values are taken from: #1 Ref.[22], #2 Ref.[13], #3 Ref.[23], #4 Ref.[18], #5 Ref.[20], #6 Ref.[24].

new record value in the field since it is nearly a factor 9 smaller than the smallest value reported in literature which equals $140\,\mu$V [22]. Using our analytical equations we are also able to compare our transducer to the most efficient microwave-to-optics converters [13, 23] today that are also based on an electro-opto-mechanical system for which no value of V_π was reported. For these devices the smallest achieved value equals around $25\,\mu$V. Different approaches of implementing a converter are much less efficient represented in a much higher value for V_π. The smallest reported values for a piezo-optomechanical, an electro-optic and a commercial system equal 24 mV [18], 260 mV [20], and 5 V (Thorlabs LN53S-FC), respectively. By minimizing the optical absorption heating leading to a smaller γ_{m} and improving η_{e} with an optimized microwave design, our device has the potential to achieve an order of magnitude lower V_π than reported here. A summary of the voltage V_π is shown for various platforms in Supplementary Fig. 11. Here, V_π is plotted against the bandwidth for which the most efficient modulation can be achieved. This is instructive to do since there is usually a dependence between these two parameters, i.e. the most efficient phase modulators use resonances leading to a limited bandwidth.

Our minimum value for the energy-per-bit E_{bit} is more than one (nearly two orders) of magnitude more efficient than for a state-of-the-art electro-optic [30] (piezo-optomechanical [18]) modulator. By improving the microwave design leading to larger η_{e} it will be possible to decrease the value of E_{bit} by nearly an order of magnitude leading to values in the sub-femtojoule range.

Important to note in these discussions is that our system requires a parametric amplification on the electro-mechanical side to achieve such low values for V_π and E_{bit} as mentioned above, i.e. we do need a microwave pump tone. This is not the case for the other approaches.

REFERENCES

[1] Lauk, N. *et al.* Perspectives on quantum transduction. *Quantum Science and Technology* **5**, 020501 (2020).
[2] Barzanjeh, S., Vitali, D., Tombesi, P. & Milburn, G. J. Entangling optical and microwave cavity modes by means of a nanomechanical resonator. *Phys. Rev. A* **84**, 042342 (2011).
[3] Marquardt, F., Chen, J. P., Clerk, A. A. & Girvin, S. M. Quantum theory of cavity-assisted sideband cooling of mechanical motion. *Phys. Rev. Lett.* **99**, 093902 (2007).
[4] Wilson-Rae, I., Nooshi, N., Zwerger, W. & Kippenberg, T. J. Theory of ground state cooling of a mechanical oscillator using dynamical back-action. *Phys. Rev. Lett.* **99**, 093901 (2007).
[5] Caves, C. M. Quantum limits on noise in linear amplifiers. *Physical Review D* **26**, 1817–1839 (1982).
[6] Weinstein, A. J. *et al.* Observation and interpretation of motional sideband asymmetry in a quantum electromechanical device. *Phys. Rev. X* **4**, 041003 (2014).
[7] Barzanjeh, S. *et al.* Stationary entangled radiation from micromechanical motion. *Nature* **570**, 480–483 (2019).
[8] Barzanjeh, S. *et al.* Mechanical on-chip microwave circulator. *Nature Communications* **8**, 953 (2017).
[9] Safavi-Naeini, A. H. *et al.* Squeezed light from a silicon micromechanical resonator. *Nature* **500**, 185–189 (2013).
[10] Gröblacher, S., Hill, J. T., Safavi-Naeini, A. H., Chan, J. & Painter, O. Highly efficient coupling from an optical fiber to a nanoscale silicon optomechanical cavity. *Appl. Phys. Lett.* **103**, 181104 (2013).
[11] Mohan, S., del Mar Hershenson, M., Boyd, S. & Lee, T. Simple accurate expressions for planar spiral inductances. *Solid-State Circuits, IEEE Journal of* **34**, 1419–1424 (1999).
[12] Zhang, R. *et al.* Integrated tuning fork nanocavity optomechanical transducers with high $f_{\mathrm{M}}Q_{\mathrm{M}}$ product and stress-engineered frequency tuning. *Applied Physics Letters* **107**, 131110 (2015).
[13] Andrews, R. W. *et al.* Bidirectional and efficient conversion between microwave and optical light. *Nature Physics* **10**, 321–326 (2014).
[14] Fink, J. M. *et al.* Quantum electromechanics on silicon nitride nanomembranes. *Nature Communications* **7**, 12396 (2016).
[15] Wollman, E. E. *et al.* Quantum squeezing of motion in a mechanical resonator. *Science* **349**, 952–955 (2015).
[16] Massel, F. *et al.* Microwave amplification with nanomechanical resonators. *Nature* **480**, 351–354 (2011).
[17] Cohen, M. A., Bothner, D., Blanter, Y. M. & Steele, G. A. Optomechanical microwave amplification without mechanical amplification. *Phys. Rev. Applied* **13**, 014028 (2020).
[18] Jiang, W. *et al.* Efficient bidirectional piezo-optomechanical transduction between microwave and optical frequency. *Nature Communications* **11**, 1166 (2020).
[19] Tsang, M. Cavity quantum electro-optics. *Physical Review A* **81**, 063837 (2010).
[20] Rueda, A., Sedlmeir, F., Kumari, M., Leuchs, G. & Schwefel, H. G. L. Resonant electro-optic frequency comb. *Nature* **568**, 378–381 (2019).
[21] Aspelmeyer, M., Kippenberg, T. J. & Marquardt, F. Cavity optomechanics. *Rev. Mod. Phys.* **86**, 1391–1452 (2014).
[22] Bagci, T. *et al.* Optical detection of radio waves through a nanomechanical transducer. *Nature* **507**, 81–85 (2014).
[23] Higginbotham, A. P. *et al.* Harnessing electro-optic correlations in an efficient mechanical converter. *Nature Physics* **14**, 1038–1042 (2018).
[24] Shao, L. *et al.* Microwave-to-optical conversion using lithium niobate thin-film acoustic resonators. *Optica* **6**, 1498–1505 (2019).
[25] Bochmann, J., Vainsencher, A., Awschalom, D. D. & Cleland, A. N. Nanomechanical coupling between microwave and optical photons. *Nature Physics* **9**, 712–716 (2013).
[26] Balram, K. C., Davanço, M. I., Song, J. D. & Srinivasan, K. Coherent coupling between radiofrequency, optical and acoustic waves in piezo-optomechanical circuits. *Nature Photonics* **10**, 346 (2016).
[27] Ramp, H. *et al.* Wavelength transduction from a 3D microwave cavity to telecom using piezoelectric optomechanical crystals. *Appl. Phys. Lett.* **116**, 174005 (2020).
[28] Rueda, A. *et al.* Efficient microwave to optical photon conversion: an electro-optical realization. *Optica* **3**, 597–604 (2016).
[29] Fan, L. *et al.* Superconducting cavity electro-optics: A platform for coherent photon conversion between superconducting and photonic circuits. *Science Advances* **4**, eaar4994 (2018).
[30] Melikyan, A. *et al.* High-speed plasmonic phase modulators. *Nature Photonics* **8**, 229–233 (2014).
