# Supplementary material for: Converting microwave and telecom photons with a silicon photonic nanomechanical interface
Source: Nat Commun. 2020 Sep 8;11:4460. doi: 10.1038/s41467-020-18269-z (PMC7479601; doi:10.1038/s41467-020-18269-z)
Supplement: Supplementary file 2 — Peer Review File [file 41467_2020_18269_MOESM2_ESM.pdf]

Editorial Note: This manuscript has been previously reviewed at another journal that is not operating a transparent peer review scheme. This document only contains reviewer comments and rebuttal letters for versions considered at Nature Communications.
